# Peroxo-Thorium(IV)-Containing Heteropolytungstates and Their Oxo-Analogues: Synthesis, Structure and Solution Studies

**DOI:** 10.1021/acs.inorgchem.5c04382

**Published:** 2026-01-08

**Authors:** Sahar Khandan, Bassem S. Bassil, Anupam Sarkar, Ayush Kant Ranga, Arnulf Materny, Samer Dawoud, Laurent Ruhlmann, Ulrich Kortz

**Affiliations:** † School of Science, 84498 Constructor University, Campus Ring 1, 28759 Bremen, Germany; ‡ UMR CNRS 7177, Laboratoire d’Electrochimie et de Chimie Physique du Corps Solide, Institut de Chimie, 27083 Université de Strasbourg 67081 Strasbourg, France

## Abstract

We report the synthesis of peroxo-thorium­(IV)-containing silico- and germanotungstates, [Th_3_(μ_3_-O_2_)­(OH)_2_(H_2_O)_3_(*A-*α-XW_9_O_34_)_2_]^12−^ (X = Si, **Th**_**3**_ **O**_**2**_ **Si**; Ge, **Th**_**3**_ **O**_**2**_ **Ge**), prepared in aqueous solution via a straightforward two-step procedure: (1) reaction of thorium­(IV) cations with trilacunary polytungstate precursors, followed by (2) the addition of hydrogen peroxide. In the absence of hydrogen peroxide, we were able to obtain the oxo-analogues, [Th_3_(μ_3_-O)­(OH)_3_(H_2_O)­(*A-*α-SiW_9_O_34_)_2_]^13–^ (**Th**_**3**_ **Si**) and [Th_3_(μ_3_-O)­(OH)_3_(*A-*α-GeW_9_O_34_)_2_]^13–^ (**Th**_**3**_ **Ge**), respectively. All four polyanions crystallized as hydrated mixed rubidium–sodium salts. Single-crystal X-ray diffraction revealed that the four polyanions **Th**_**3**_ **O**_**2**_ **Si**, **Th**_**3**_ **O**_**2**_ **Ge**, **Th**_**3**_ **Si**, and **Th**_**3**_ **Ge** consist of two {*A-*α-XW_9_O_34_} fragments sandwiching a central, triangular thorium core. The structures of **Th**_**3**_ **O**_**2**_ **X** (X = Si, Ge) comprise a central peroxo-group with a rare side-on μ_3_-coordination mode between the three thorium centers, representing the first example of peroxo-thorium­(IV)-containing polyoxotungstates. Furthermore, ^183^W NMR and electrochemical studies in solution were performed.

## Graphical abstract

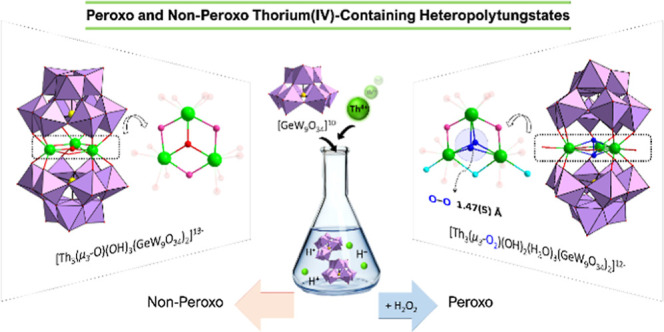

## Introduction

Polyoxometalates (POMs) are discrete, anionic polynuclear metal-oxo complexes comprising early *d*-block metal addenda in high oxidation states such as tungsten­(VI), molybdenum­(VI) or vanadium­(V), which are frequently 6-coordinated by oxo ligands. POMs exhibit a remarkable structural and compositional diversity and manifold physicochemical properties.^1^ As a result, POMs are of academic and applied interest in many different areas, such as homogeneous and heterogeneous catalysts,^2^ electron transfer mediators,^3^ or models for elucidating fundamental principles of molecular reactivity.^4^

Peroxo-functionalized POMs (peroxo-POMs) represent a distinct subclass of POM chemistry. The first incorporation of peroxo groups into POM frameworks was reported in 1969 by Beiles et al.^5^ In 1985, Venturello and co-workers described the synthesis and crystal structure of [PO_4_{WO­(O_2_)_2_}_4_]^3–^, commonly referred to as the “Venturello ion”, which has since garnered significant attention for its unique and versatile reactivity in hydrogen peroxide-based oxidation reactions.^6^ In fact, the presence of peroxo-ligands distinguishes the Venturello ion from traditional POMs, imparting distinctive catalytic properties to this species. Subsequent research allowed to introduce peroxo groups onto secondary addenda metal centers, in particular niobium­(V), allowing for precise control over the composition and functionality for specific applications. For example, Finke’s group first introduced peroxo-niobium heteropolytungstates in 1991 by presenting the formation of [(NbO_2_)_3_SiW_9_O_37_]^7–^ and its catalytic activity in organic phase olefin epoxidation.^7^ In 2001, Hill’s group reported on the synthesis and analysis of α_1_- and α_2_-[(NbO_2_)­P_2_W_17_O_61_]^7–^ polyanions, highlighting their potent inhibitory activity against HIV-1 protease.^8^

Our group has investigated the synthesis and characterization of various peroxo-polytungstates by the reaction of tetravalent metal ions, particularly zirconium­(IV), hafnium­(IV), and cerium­(IV) with lacunary POM precursors in the presence of hydrogen peroxide.^9^ For instance, in 2008 the isostructural polyanions [M_6_(O_2_)_6_(OH)_6_(γ-SiW_10_O_36_)_3_]^18–^ (M = Zr^IV^, Hf^IV^) were reported, comprising a crown-shaped peroxo-zirconium/hafnium core, {Zr_6_(O_2_)_6_(OH)_6_}, stabilized by three dilacunary {γ-SiW_10_} Keggin units.^9a^ In 2019, the peroxo-cerium­(IV)-containing [Ce_6_(O_2_)_9_(GeW_10_O_37_)_3_]^24–^ was reported, which proved to be a recyclable homogeneous oxidation catalyst in sulfoxidation.^9c^ This was followed by the isolation of the dimeric peroxo-zirconium/hafnium-incorporated Wells-Dawson polyanions, [M_2_(O_2_)_2_(X_2_W_17_O_61_)_2_]^16–^ (M = Zr^IV^, Hf^IV^; X = P^V^, As^V^), which exhibited good homogeneous, heterogeneous and biphasic catalytic performance in various peroxide-based oxidations.^9d^ Recently, we synthesized and structurally characterized a novel peroxo-bridged dicerium­(IV)-dilithium polyoxometalate, [(Ce_2_O_2_)­Li_2_(P_2_W_16_O_59_)_2_]^16–^. This polyanion features a side-on peroxo bridge connecting two cerium­(IV) and two Li^+^ ions, embedded between two {P_2_W_16_} Dawson-type fragments, each bearing a vacant site in the equatorial belt.^9e^

The actinide metal cation thorium­(IV) has very similar chemical properties with its lanthanide counterpart cerium­(IV) in the same group. Both cations share similarities in coordination chemistry, oxidation states, ionic radii, and strong affinity for oxygen-donor ligands. Although early research primarily explored thorium­(IV) complexes with organic ligands,^10^ studies involving thorium­(IV) interactions with inorganic lacunary polytungstate species remain limited.

One of the earliest structurally characterized examples of thorium­(IV)-incorporated polytungstates is the sodium salt of [ThW_10_O_36_]^8–^.^11^ The compound demonstrated the ability of the two {W_5_O_18_} moieties to accommodate thorium­(IV) ions through coordination to terminal oxygen atoms.^12^ In 2003, Pope and co-workers reported the isolation of the first thorium­(IV)-containing heteopolytungstate [Th­(P_2_W_17_O_61_)_2_]^16–^, representing the possibility for the complexation of thorium­(IV) ions with monolacunary Wells-Dawson polytungstates.^13^ Later on, Duval’s group further investigated the bonding interactions of thorium­(IV) with lacunary Keggin-type POMs. Their findings revealed the formation and isolation of the polyanion [Th_3_(μ_3_-O)­(OH)_3_(SiW_9_O_34_)_2_]^13–^.^14^ However, powder X-ray diffraction and IR spectroscopic analyses indicated the presence of crystalline impurities, attributed to a polymeric thorium-acetate species. Thereafter, in 2019, the same group introduced the molecular structure of the polyanion [K_4_{Th_3_(H_2_O)_3_{AsO­(μ_2_-O)_3_}_2_}­{Th_3_(H_2_O)_2_{AsO­(μ_2_-O)_3_}_2_}_3_(AsW_10_O_38_)_6_]^38–^, indicating the formation of a large polyanionic system stabilized by thorium­(IV).^15^ Following these developments, our group recently reported a thorium-centered polyoxopalladate­(POP)-based metal–organic framework, [{Th^IV^Pd^II^_12_O_8_(CPA)_8_}­Ba_6_] (CPA = *p*-carboxyphenylarsonate), demonstrating further the versatile coordination chemistry of thorium ions within POP-based assemblies.^16^

To date, no peroxo-thorium­(IV)-containing POM has been isolated. Hence, we decided to investigate the interaction of thorium­(IV) ions with lacunary heteropolytungstates in the presence of hydrogen peroxide.

## Experimental Section

### Materials and Methods

Caution! Despite the low specific activity and long half-life (1.4 × 10^10^ years) of ^232^Th, it is essential to strictly adhere to standard safety protocols for handling radioactive materials when working with the quantities used in the following syntheses.

All reagents and chemicals were commercially available and used without additional purification. The precursor salt Na_10_[*A-*α*-*SiW_9_O_34_]·18H_2_O and Na_10_[*A-*α*-*GeW_9_O_34_]·18H_2_O were prepared according to the published procedures in the literature and purity was confirmed by infrared spectroscopy (Figures S1 and S2).^17^ The Fourier transform infrared (FT-IR) spectra were obtained on a SHIMADZU IRSpirit spectrometer (4000–400 cm^–1^) using KBr pellets. Thermogravimetric analysis (TGA) was conducted on a TA Instruments Model SDT Q600 thermobalance, operating under a constant flow of N_2_ gas (Figures S3–S6). The temperature gradually increased from room temperature to 600 °C at a rate of 5 °C/min. The ^183^W NMR spectra were recorded at room temperature on JEOL ECP 400 instrument equipped with a 10 mm probe and the chemical shifts are reported with respect to 1 M Na_2_WO_4_ in H_2_O. The ^29^Si NMR spectra were recorded on the same instrument, equipped with a 5 mm probe. The samples were dissolved in water and the spectra were acquired overnight at room temperature. The chemical shifts are reported relative to tetramethylsilane (TMS) as reference. The UV–vis spectra were recorded on a Varian Cary 100 Bio UV–vis spectrophotometer using quartz cuvettes. Elemental analysis was carried out at Zentrallabor, Technische Universität Hamburg, Am Schwarzenberg-Campus 1, 21073 Hamburg. The sodium analyses were performed by atomic absorption in-house on a Varian SpectrAA 220 spectrometer.

### Rb_9_Na_3_[Th_3_(μ_3_-O_2_)­(OH)_2_(H_2_O)_3_(*A-*α-SiW_9_O_34_)_2_]·10H_2_O (RbNa–Th_3_O_2_Si)

A solid sample of Th­(NO_3_)_4_·4H_2_O (0.098 g, 0.177 mmol) was dissolved in 20 mL 0.1 M RbCl aqueous solution. Subsequently, a solid sample of Na_10_[*A-*α*-*SiW_9_O_34_]·18H_2_O (**Na–SiW**_**9**_) (0.492 g, 0.177 mmol) was added to this solution and the mixture was stirred at 55 °C for 10 min. The pH of the resulting solution was adjusted to 4 using 1 M HCl, followed by stirring for an additional 30 min at the same temperature. Then, 1 mL of 30% hydrogen peroxide was added and the solution was stirred continuously for another 30 min. This reaction mixture was allowed to cool down to room temperature in an open vial. Light-yellow crystals started to form within 1–2 days and were subsequently collected by filtration and then air-dried. Isolated yield: 0.204 g (37%, based on Th). FT-IR (1% KBr disk/cm^–1^): 3452 (br), 1630 (s), 1002 (m), 939 (m), 890 (s), 776 (s), 680 (w), 543 (sh), 514 (w) 464 (sh), see Figure S1. Elemental analysis (%): calcd Rb 12.2, Na 1.0, Th 11.1, Si 0.9, W 52.6; found, Rb 12.3, Na 0.9, Th 11.7, Si 0.9, W 52.3.

### Rb_10_Na_2_[Th_3_(μ_3_-O_2_)­(OH)_2_(H_2_O)_3_(*A-*α-GeW_9_O_34_)_2_]·7H_2_O (RbNa–Th_3_O_2_Ge)

The synthesis of this compound was identical to that of **RbNa–Th**_**3**_ **O**_**2**_ **Si**, but Na_10_[*A-*α*-*GeW_9_O_34_]·18H_2_O (**Na-GeW**_**9**_) (0.500 g, 0.177 mmol) was used instead of **Na–SiW**_**9**_. Light-yellow crystals started to form within 1–2 days and were subsequently collected by filtration and then air-dried. Isolated yield: 0.220 g (39%, based on Th). FT-IR (1% KBr disk/cm^–1^): 3466 (br), 1621 (s), 938 (m), 889 (m), 787 (s), 670 (m), 518 (w), 472 (w), 425 (w) (Figure S2). Elemental analysis (%): calcd Rb 13.4, Na 0.7, Th 10.9, Ge 2.3, W 51.8; found, Rb 13.6, Na 0.6, Th 10.8, Ge 2.3, W 52.1.

### Rb_9_Na_4_[Th_3_(μ_3_-O)­(OH)_3_(H_2_O)­(*A-*α-SiW_9_O_34_)_2_]·20H_2_O (RbNa–Th_3_Si)

A solid sample of Th­(NO_3_)_4_·4H_2_O (0.098 g, 0.265 mmol) was dissolved in 20 mL 0.1 M RbCl aqueous solution. Then a solid sample of **Na–SiW**_**9**_ (0.492 g, 0.177 mmol) was added to this solution, and the mixture was stirred at 55 °C for 10 min. Subsequently, the pH was adjusted to 4 with 1 M HCl, followed by stirring at 55 °C for 1 h. The reaction mixture was then allowed to cool to room temperature, and a white crystalline product formed within 1–3 days, which was collected by filtration and then air-dried. Isolated yield: 0.234 g (41%, based on Th). FT-IR (1% KBr disk/cm^–1^): 3431 (br), 1631 (s), 985 (sh), 946 (m), 876 (s), 827 (w), 775 (s), 692 (m), 529 (m), 425 (w), see Figure S1. Elemental analysis (%): calcd Rb 12.0, Na 1.4, Th 10.8, Si 0.9, W 51.3; found, Rb 12.4, Na 1.2, Th 11.0, Si 0.9, W 51.2.

### Rb_9_Na_4_[Th_3_(μ_3_-O)­(OH)_3_(*A-*α-GeW_9_O_34_)_2_]·15H_2_O (RbNa–Th_3_Ge)

The synthesis of this compound was identical to that of **RbNa–Th**_**3**_ **Si**, but **Na-GeW**_**9**_ (0.500 g, 0.177 mmol) was used instead of **Na–SiW**_**9**_. A white crystalline product formed within 1–3 days, which was collected by filtration and then air-dried. Isolated yield: 0.210 g (37%, based on Th). FT-IR (1% KBr disk/cm^–1^): 3413 (br), 1623 (s), 944 (m), 888 (m), 779 (s), 695 (m), 518 (w), 459 (w), 421 (w) (Figure S2). Elemental analysis (%): calcd. Rb 12.0, Na 1.4, Th 10.8, Ge 2.2, W 51.4; found Rb 11.8, Na 1.3, Th 10.9, Ge 2.2, W 51.8.

All four polyanions described above can also be isolated as mixed potassium–sodium salts by substituting the 0.1 M RbCl solution by 0.5 M KCl as the solvent (see the FT-IR spectra of the potassium–sodium salts of all polyanions in Figure S7).

### Single-Crystal X-ray Diffraction

Data acquisition was performed on a Rigaku XtaLAB Synergy single-crystal diffractometer, configured with Dualflex and HyPix detection systems, and employing kappa geometry. The instrument utilized a graphite monochromator with a MoKα radiation source (λ = 0.71073 Å). The collection of crystallographic data was facilitated by the CrysAlisPro software package.^18^ Crystals were mounted on Hampton cryoloops using Paratone-N oil and measured at 100 K. An empirical absorption correction was applied using the ABSPACK program to account for absorption effects.^19^ An initial structure solution was achieved through direct methods, followed by iterative refinements using successive difference-Fourier maps. Final structural refinements were carried out using the SHELXL-2014 software,^20^ employing a full-matrix least-squares approach. These refinements were conducted against the absolute values of the structure factor |*F*|, incorporating all collected data to ensure comprehensive analysis. Crystal structure illustrations were generated using diamond, version 3.2 (Crystal Impact GbR). Crystallographic data for all compounds are summarized in Table 1. Further details on the crystal structure investigations may be obtained free of charge under CCDC 2472475 (**RbNa–Th**_**3**_ **O**_**2**_ **Si**), 2472473 (**RbNa–Th**_**3**_ **O**_**2**_ **Ge**), 2472474 (**RbNa–Th**_**3**_ **Si**), and 2472472 (**RbNa–Th**_**3**_ **Ge**) from The Cambridge Crystallographic Data Centre via http://www.ccdc.cam.ac.uk/data_request/cif.

**Table 1:** Crystallographic Data for **RbNa–Th**_**3**_ **O**_**2**_ **Si**, **RbNa–Th**_**3**_ **O**_**2**_ **Ge**, **RbNa–Th**_**3**_ **Si**, and **RbNa–Th**_**3**_ **Ge**

	**RbNa–Th_3_O_2_Si**	**RbNa–Th_3_O_2_Ge**	**RbNa–Th_3_Si**	**RbNa–Th_3_Ge**
formula^a^	Rb_9_Na_3_[Th_3_(O_2_)(OH)_2_(H_2_O)_3_(SiW_9_O_34_)_2_]·10H_2_O	Rb_10_Na_2_[Th_3_(O_2_)(OH)_2_(H_2_O)_3_(GeW_9_O_34_)_2_]·7H_2_O	Rb_9_Na_4_[Th_3_(O)(OH)_3_(H_2_O)(SiW_9_O_34_)_2_]·20H_2_O	Rb_9_Na_4_[Th_3_(O)(OH)_3_(GeW_9_O_34_)_2_]·15H_2_O
formula weight^a^ (gm/mol)	6233.97	6385.46	6453.27	6437.05
crystal system	Triclinic	Triclinic	Monoclinic	Monoclinic
space group	*P*1̅	*P*1̅	*I*2/*a*	*I*2/*m*
*a* (Å)	13.1673 (1)	13.1634 (5)	20.0503 (2)	25.3188 (4)
*b* (Å)	16.9663 (2)	16.9010 (6)	36.3617 (3)	15.7717 (1)
*c* (Å)	22.0219 (2)	22.1373 (8)	23.6336 (2)	26.7824 (4)
α (°)	103.599 (1)	103.363 (2)	90	90
β (°)	98.763 (1)	99.102 (2)	94.689 (1)	115.133 (2)
γ (°)	109.734 (1)	109.8049 (19)	90	90
volume (Å^3^)	4354.16 (8)	4355.2 (3)	17172.7 (3)	9682.2 (3)
*Z*	2	2	8	4
D _calc_ (Mg/m^3^)	4.755	4.869	4.918	4.441
absorption coefficient (mm^–1^)	33.92	35.11	34.68	30.85
crystal size(mm)	0.19 × 0.13 × 0.05	0.16 × 0.10 × 0.05	0.16 × 0.14 × 0.04	0.18 × 0.09 × 0.04
F(000)	5348	5472	21912	11196
reflections used [*I* > 2σ (*I*)]	24854	14351	24196	15281
independent reflections	32612	17797	31774	18455
*R*_int_	0.155	0.117	0.114	0.066
goodness-of-fit on F^2^	1.003	1.005	1.005	1.000
*R*_1_[I > 2σ (I)]^b^	0.052	0.047	0.046	0.093
w*R*_2_ ^c^ (all data)^c^	0.146	0.128	0.122	0.221

a The entries are the actual formula units and weights as obtained from elemental analysis on bulk samples.

b *R*_1_ = Σ||*F*_0_| – |*F*_c_||/Σ|*F*_0_|.

c *wR*_2_ = [Σ*w* (*F*_0_^2^ – *F*_c_^2^)^2^/Σ*w* (*F*_0_^2^)^2^]^1/2^.

### Raman Spectroscopy

Raman spectra on the novel compounds were recorded using a B&W Tek i-Raman Plus compact spectrometer, which is equipped with a fiber-optic probe for excitation and signal collection, as well as a charge-coupled device (CCD) detector operating within the spectral range of 65-3350 cm^–1^. The system features a built-in near-infrared laser (785 nm) and a fixed low-groove-density grating, enabling spectral acquisition over a broad range (up to 3350 cm^–1^) in a single detection window. Additionally, the laser’s optical fiber can be connected to a collimator tube, facilitating its integration with a microscope system for detailed sample observation and analysis via a microscope objective. A 50× magnification objective was employed, yielding a beam spot size of approximately 30–35 μm. The spectral resolution of the Raman system was approximately 4 cm^–1^. Wavenumber calibration was verified using the breathing mode of toluene at 1003.7 cm^–1^. All Raman spectra were recorded using a 785 nm diode laser at a power of 100 mW, covering the spectral range of 1200–400 cm^–1^, with each measurement repeated three times per spectral window. Solid-state Raman spectra were recorded with an exposure time of 4 s. For solution-phase measurements, 10 mM solutions were prepared by dissolving the compounds in deionized water, followed by sonication in an ultrasonic bath for 10 min. The exposure time for solution samples was set to 16 s.

### Electrochemistry

Voltammetric data have been recorded with a standard three-electrode system using an Metrohm Autolab PGSTAT30 potentiostat using a conventional three-electrode setup. Potentials are quoted against a saturated calomel electrode (SCE). The counter electrode was a platinum gauze of large surface area. All experiments were performed at room temperature. The working electrode was glassy carbon electrode (GC, Ø = 3 mm), the counter electrode was platinum wire, and the reference electrode was a SCE electrode connected through a salt bridge. The working cell was surrounded by a grounded Faraday cage and all studies were carried out at room temperature and under an argon flow. Ultrapure water (Millipore, 18.2 MΩ·cm^–1^, 25 °C) was used to prepare all electrolyte solutions. The composition and pH of the media used for both the electrochemical experiments and the stability studies by spectrophotometry were as follows: 0.5 M Na_2_SO_4_ + H_2_SO_4_ (pH 2 and pH 4). The solutions were deaerated thoroughly by bubbling argon through the solution and kept under argon atmosphere during the whole experiment. The glassy carbon electrode (GC) was cleaned before each measurement according to the following procedure: polishing on a microcloth polishing pad with diamond paste (DP-paste M, 1, 3, and 6 μm particle sizes); washing with ultrapure water; sonication in an ultrasonic bath for 5 min.

## Results and Discussion

### Synthesis and Structure

We have synthesized the peroxo-thorium­(IV)-containing silico- and germanotungstates, [Th_3_(μ_3_-O_2_)­(OH)_2_(H_2_O)_3_(*A*-α-SiW_9_O_34_)_2_]^12–^ (**Th**_**3**_ **O**_**2**_ **Si**) and [Th_3_(μ_3_-O_2_)­(OH)_2_(H_2_O)_3_(*A*-α-GeW_9_O_34_)_2_]^12–^ (**Th**_**3**_ **O**_**2**_ **Ge**), respectively, by the reaction of thorium­(IV) ions with the respective trilacunary POM precursors in acidic aqueous solution in the presence of hydrogen peroxide. In the absence of hydrogen peroxide the corresponding oxo-analogues [Th_3_(μ_3_-O)­(OH)_3_(H_2_O)­(*A*-α-SiW_9_O_34_)_2_]^13–^ (**Th**_**3**_ **Si**) and [Th_3_(μ_3_-O)­(OH)_3_(*A*-α-GeW_9_O_34_)_2_]^13–^ (**Th**_**3**_ **Ge**), respectively, were obtained. All four polyoxoanions were isolated as hydrated mixed rubidium–sodium salts, Rb_9_Na_3_[Th_3_(μ_3_-O_2_)­(OH)_2_(H_2_O)_3_(*A-*α-SiW_9_O_34_)_2_]·10H_2_O (**RbNa–Th**_**3**_ **O**_**2**_ **Si**), Rb_10_Na_2_[Th_3_(μ_3_-O_2_)­(OH)_2_(H_2_O)_3_(*A-*α-GeW_9_O_34_)_2_]·7H_2_O (**RbNa–Th**_**3**_ **O**_**2**_ **Ge**), Rb_9_Na_4_[Th_3_(μ_3_-O)­(OH)_3_(H_2_O)­(*A-*α-SiW_9_O_34_)_2_]·20H_2_O (**RbNa–Th**_**3**_ **Si**) and Rb_9_Na_4_[Th_3_(μ_3_-O)­(OH)_3_(*A-*α-GeW_9_O_34_)_2_]·15H_2_O (**RbNa–Th**_**3**_ **Ge**), respectively.

Single-crystal X-ray diffraction analysis on the mixed rubidium–sodium salts of the peroxo-thorium­(IV)-containing heteropolytungstates [Th_3_(μ_3_-O_2_)­(OH)_2_(H_2_O)_3_(*A-*α-XW_9_O_34_)_2_]^12–^ (X = Si, **Th**_**3**_ **O**_**2**_ **Si**; Ge, **Th**_**3**_ **O**_**2**_ **Ge**) revealed that both compounds are isomorphous and they crystallize in the triclinic space group *P*1̅ (Table 1). The polyanions exhibit a distinct sandwich-type POM architecture, comprising a central, cationic [Th_3_(μ_3_-O_2_)­(OH)_2_(H_2_O)_3_]^8+^ unit encapsulated by two trilacunary [XW_9_O_34_]^10–^ (X = Si, Ge) fragments (Figure 1). Within the central unit the three thorium­(IV) guest ions are coordinated via a rare side-on μ_3_-peroxo bridge, a coordination motif that is unprecedented for peroxo-POMs. A comparable μ_3_–η^2^: η^2^ *:*η^2^ peroxo-coordination has previously been reported for the extended structure of Th­(O_2_)­(SO_4_)­(H_2_O)_2_ polymeric chains.^21^ The O–O bond lengths in the peroxo units of **Th**_**3**_ **O**_**2**_ **Si** and **Th**_**3**_ **O**_**2**_ **Ge** are 1.50(6) Å and 1.47(5) Å, respectively, closely aligning with reported values for the μ_3_-bridged peroxo-thorium in Th­(O_2_)­(SO_4_)­(H_2_O)_2_ (1.48(6) Å).^21^ These values also fall within the range of reported μ_2_-bridged peroxo-POM motifs discussed in the Introduction section.

**Figure 1:**
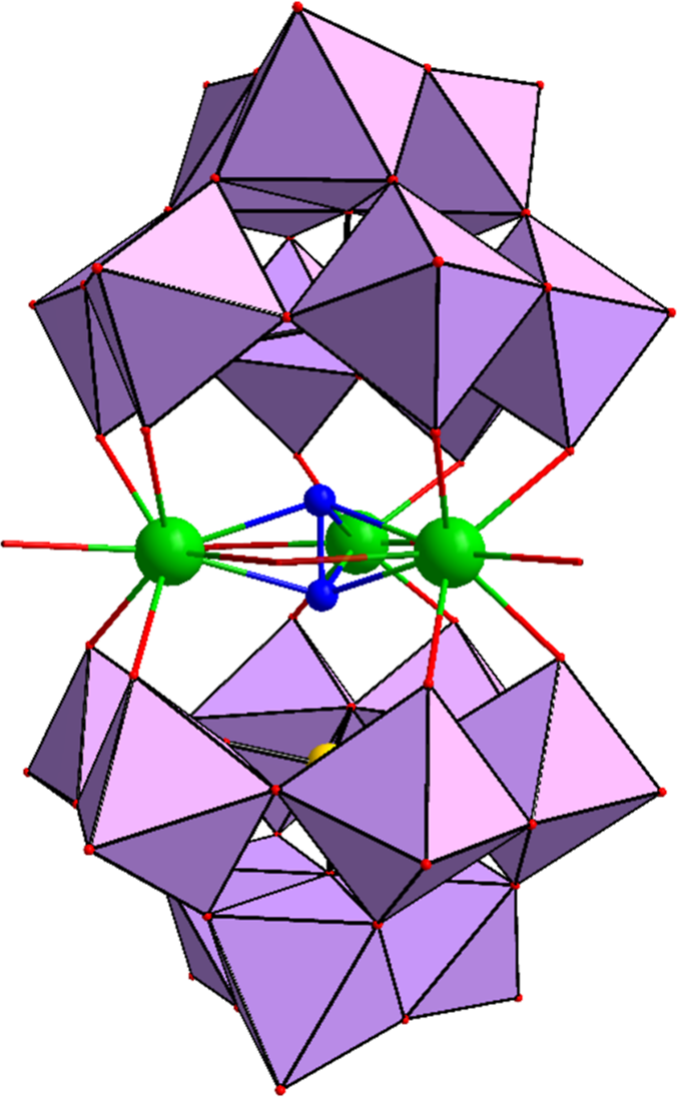
Combined polyhedral/ball-and-stick representation of [Th_3_(μ_3_-O_2_)­(OH)_2_(H_2_O)_3_(XW_9_O_34_)_2_]^12–^ (X = Si, Ge). Color code: Th (green), WO_6_ octahedra (purple), Ge/Si (yellow), O (red). For clarity, the peroxo oxygens are highlighted in dark blue.

As detailed in Figure 2, each thorium­(IV) center is coordinated from each side by two oxo-ligands from two edge-shared {WO_6_} octahedra belonging to a “diad” of one {XW_9_} Keggin unit. The atom Th1 is 8-coordinated by four more oxo-ligands, two belonging to the central μ_3_-peroxo bridge, and two protonated (hydroxo) bridges to Th2 and Th3 within the central unit. On the other hand, Th2 and Th3 are each 9-coordinated by the peroxo and hydroxo bridges shared with Th1, in addition to a Th2-(H_2_O)–Th3 aqua bridge and a terminal aqua ligand. Bond valence sum (BVS) calculations confirm the +4 oxidation state for the thorium centers and the protonation within the [Th_3_(μ_3_-O_2_)­(OH)_2_(H_2_O)_3_]^8+^ core (see Tables S1 and S2).^22^ The difference in coordination number, in addition to the longer Th2–(H_2_O)–Th3 bond length as compared to Th1–(OH)–Th2/3 break the symmetry in polyanions **Th**_**3**_ **O**_**2**_ **Si** and **Th**_**3**_ **O**_**2**_ **Ge**, resulting in idealized C_*2v*_ point group symmetry in the solid-state.

**Figure 2:**
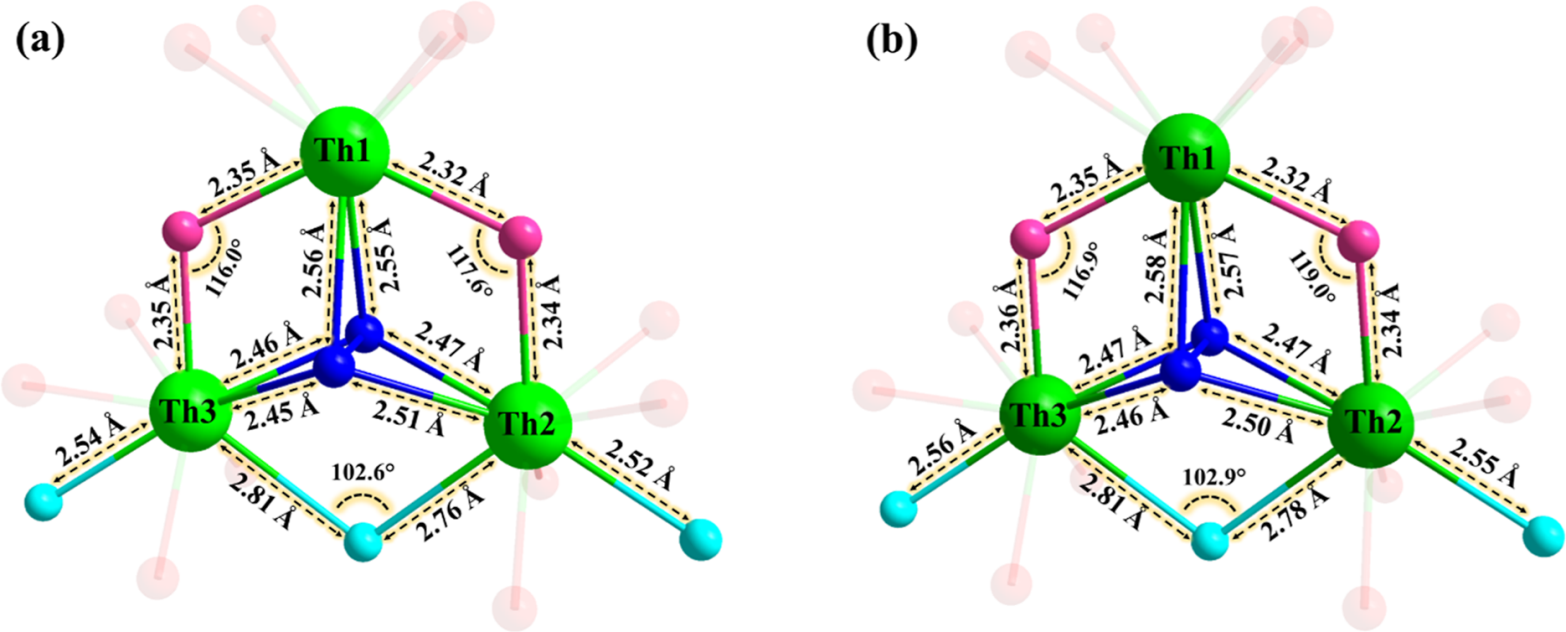
Ball-and-stick representations of the central {Th_3_(μ_3_-O_2_)­(OH)_2_(H_2_O)_3_} core in polyanions (a) **Th**_**3**_ **O**_**2**_ **Si** and (b) **Th**_**3**_ **O**_**2**_ **Ge**. Color code: Th (green), peroxo (dark blue), hydroxo (pink), and aqua (turquoise). The oxygen atoms bonded to the polyanion framework are displayed with reduced visual intensity for better emphasis on the central core. The bond lengths are indicated with two decimals (see cif files for details).

In the absence of hydrogen peroxide the peroxo-free analogues [Th_3_(μ_3_-O)­(OH)_3_(H_2_O)­(*A-*α-SiW_9_O_34_)_2_]^13–^ (**Th**_**3**_ **Si**) and [Th_3_(μ_3_-O)­(OH)_3_(*A-*α-GeW_9_O_34_)_2_]^13–^ (**Th**_**3**_ **Ge**), which also crystallize as mixed rubidium–sodium salts, albeit in the respective monoclinic space groups *I*2/*a* and *I*2/*m*. As depicted in Figure 3 and based on single-crystal XRD analysis, a μ_3_-oxo bridge (as confirmed by BVS calculations) replaces the peroxo group as the central ligand of all three thorium­(IV) centers in **Th**_**3**_ **Si** and **Th**_**3**_ **Ge**.

**Figure 3:**
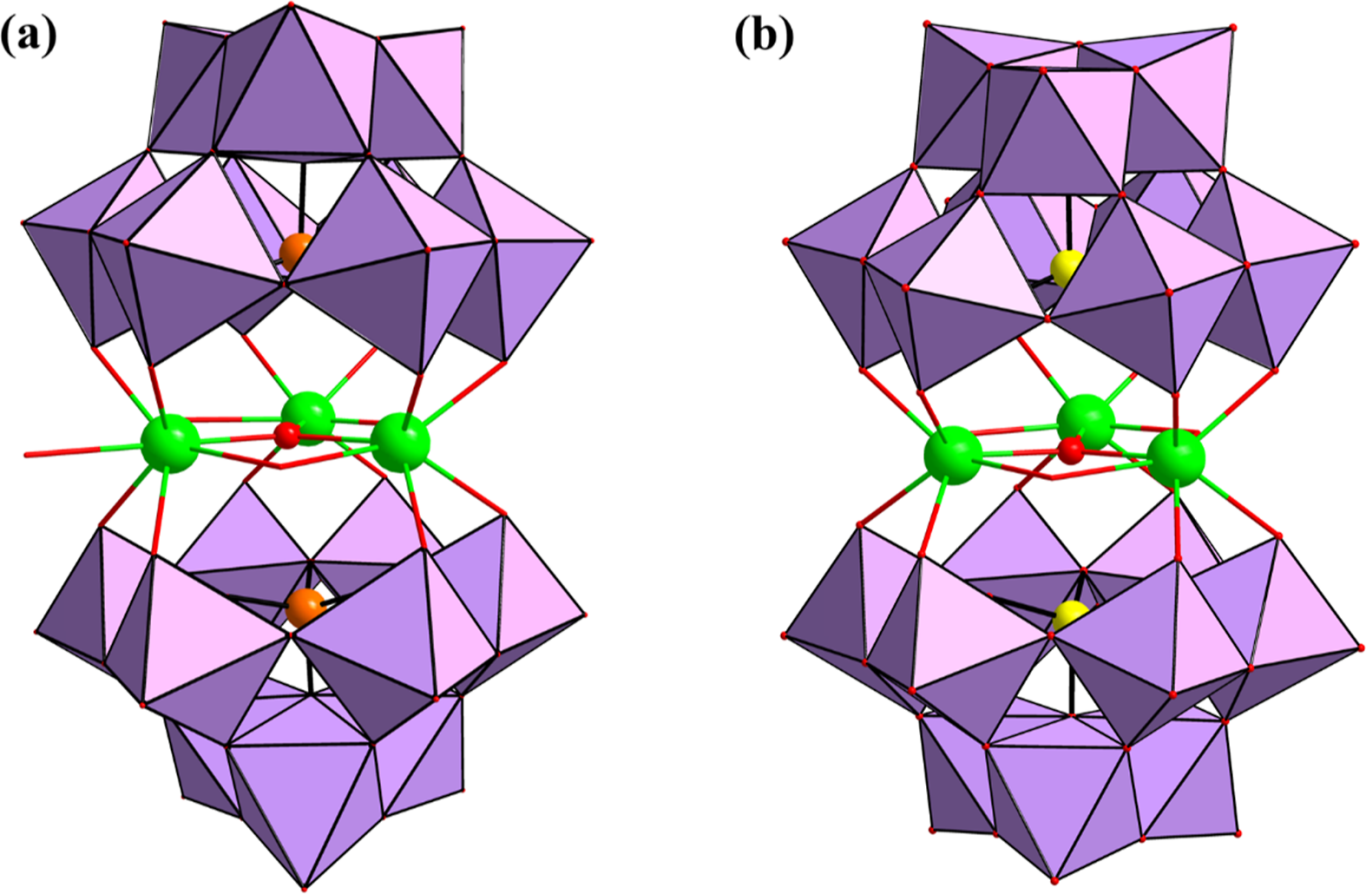
Combined polyhedral/ball-and-stick representation of (a) [Th_3_(μ_3_-O)­(OH)_3_(H_2_O)­(SiW_9_O_34_)_2_]^13–^ (**Th**_**3**_ **Si**) and (b) [Th_3_(μ_3_-O)­(OH)_3_(GeW_9_O_34_)_2_]^13–^ (**Th**_**3**_ **Ge**). Color code: Th (green), WO_6_ octahedra (purple), Si (orange), Ge (yellow), and O (red).

In **Th**_**3**_ **Si** the central cationic unit [Th_3_(μ_3_-O)­(OH)_3_(H_2_O)]^7+^ exhibits Th–OH–Th bond distances between Th2–Th3 and Th1–Th3, which are slightly longer than those between Th1–Th2, due to the terminal aqua ligand on Th3. This asymmetry in bonding results in an overall point group symmetry of C_*2v*_ for **Th**_**3**_ **Si**. On the other hand, for **Th**_**3**_ **Ge** all Th–OH–Th bond lengths in the central cationic core [Th_3_(μ_3_-O)­(OH)_3_]^7+^ are approximately equal (ca. 2.35–2.39 Å), as the coordination number and geometry is the same for all three thorium ions (no aqua ligand present), resulting in a symmetric arrangement and an overall D_*3h*_ point group symmetry. All oxo protonation modes and thorium oxidation states were supported by BVS calculations (Figure 4, Tables S3 and S4).

**Figure 4:**
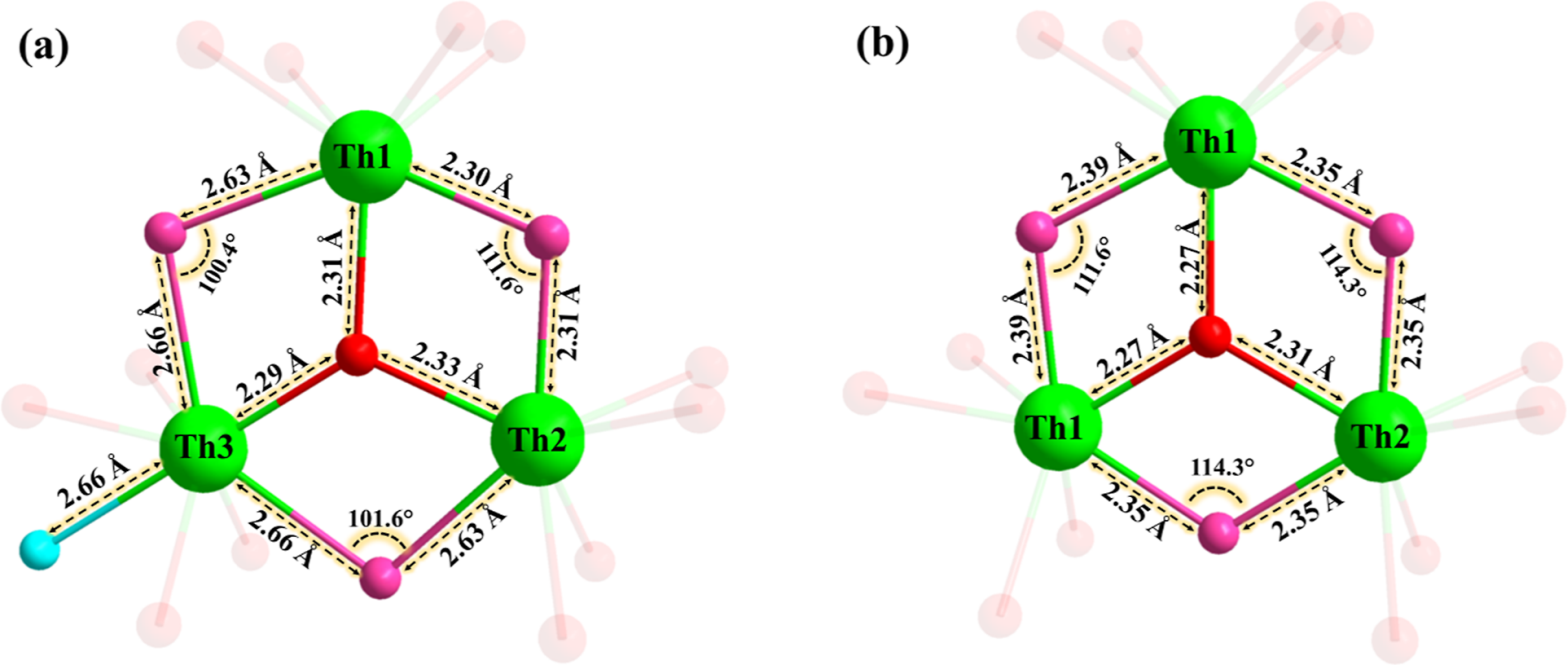
Ball-and-stick representation of the central (a) {Th_3_(μ_3_-O)­(OH)_3_(H_2_O)} unit in polyanion **Th**_**3**_ **Si** and (b) {Th_3_(μ_3_-O)­(OH)_3_} unit in polyanion **Th**_**3**_ **Ge**. Color code: Th (green), oxo (red), hydroxo (pink), and aqua (turquoise). The oxygen atoms bonded to the polyoxoanion framework are displayed with reduced visual intensity for better emphasis on the central core. The bond lengths are indicated with two decimals (see cif files for details).

The peroxo-POMs **Th**_**3**_ **O**_**2**_ **Ge** and **Th**_**3**_ **O**_**2**_ **Si** were prepared by the reaction of Th­(NO_3_)_4_ with the respective trilacunary Keggin precursor salts **Na–SiW**_**9**_ or **Na-GeW**_**9**_ in a molar ratio of 1:1 in 0.1 M RbCl solution at pH 4. These are optimized conditions which allowed for the isolation of pure crystalline material in good yield. Changes in pH as well as the molar ratio led either to the formation of a product mixture or to a lower yield. The synthesis follows a two-step procedure under mild heating, where hydrogen peroxide is added in the second step. The corresponding oxo-analogues **Th**_**3**_ **Ge** and **Th**_**3**_ **Si** were obtained by the same synthetic procedures, but in the absence of hydrogen peroxide. Based on these observations, the first step involves the formation of the sandwich-type POM structure, followed by peroxo-insertion upon addition of hydrogen peroxide in the second step. We could also isolate mixed potassium–sodium salts of the four polyanions by using 0.5 M KCl as reaction medium, but only as polycrystalline material. The FT-IR spectra of the potassium–sodium and the rubidium–sodium salts are superimposable, see Figure S7.

### FT-IR Spectroscopy

The FT-IR spectra in the 400–1100 cm^–1^ POM “fingerprint region” of the four novel compounds and the corresponding lacunary Keggin precursors are depicted in Figure 5. Primarily, the structural transformation from the lacunary Keggin ions to the thorium-containing sandwich compounds is reflected by significant spectral differences for **Na–XW**_**9**_ on the one hand and for **RbNa–Th**_**3**_ **O**_**2**_ **X** and **RbNa–Th**_**3**_ **X** (X = Si, Ge) on the other hand. Moreover, a comparison of the spectra for the silico-versus germanotungstate containing compounds reveals changes in the vibrational frequencies of characteristic stretching modes, which can be attributed to significant differences in the X–O bond lengths (Si vs Ge).^23^ We also observed that overall the FT-IR spectra of the peroxo-containing compounds exhibit a pattern of characteristic peaks closely resembling those of the nonperoxo analogues. A spectral comparison of **Th**_**3**_ **O**_**2**_ **Si** and **Th**_**3**_ **Si** reveals only subtle shifts in the vibrational frequencies and a shoulder peak at 827 cm^–1^ for **Th**_**3**_ **Si**, which is absent in the spectrum of **Th**_**3**_ **O**_**2**_ **Si**, reflecting only minor rearrangements in the Th_3_ core upon loss of the peroxo moiety. In a similar fashion, the IR spectra of the **Th**_**3**_ **O**_**2**_ **Ge** and **Th**_**3**_ **Ge** display essentially identical vibrational profiles, though with discernible wavenumber shifts compared to the silicon analogues.

**Figure 5:**
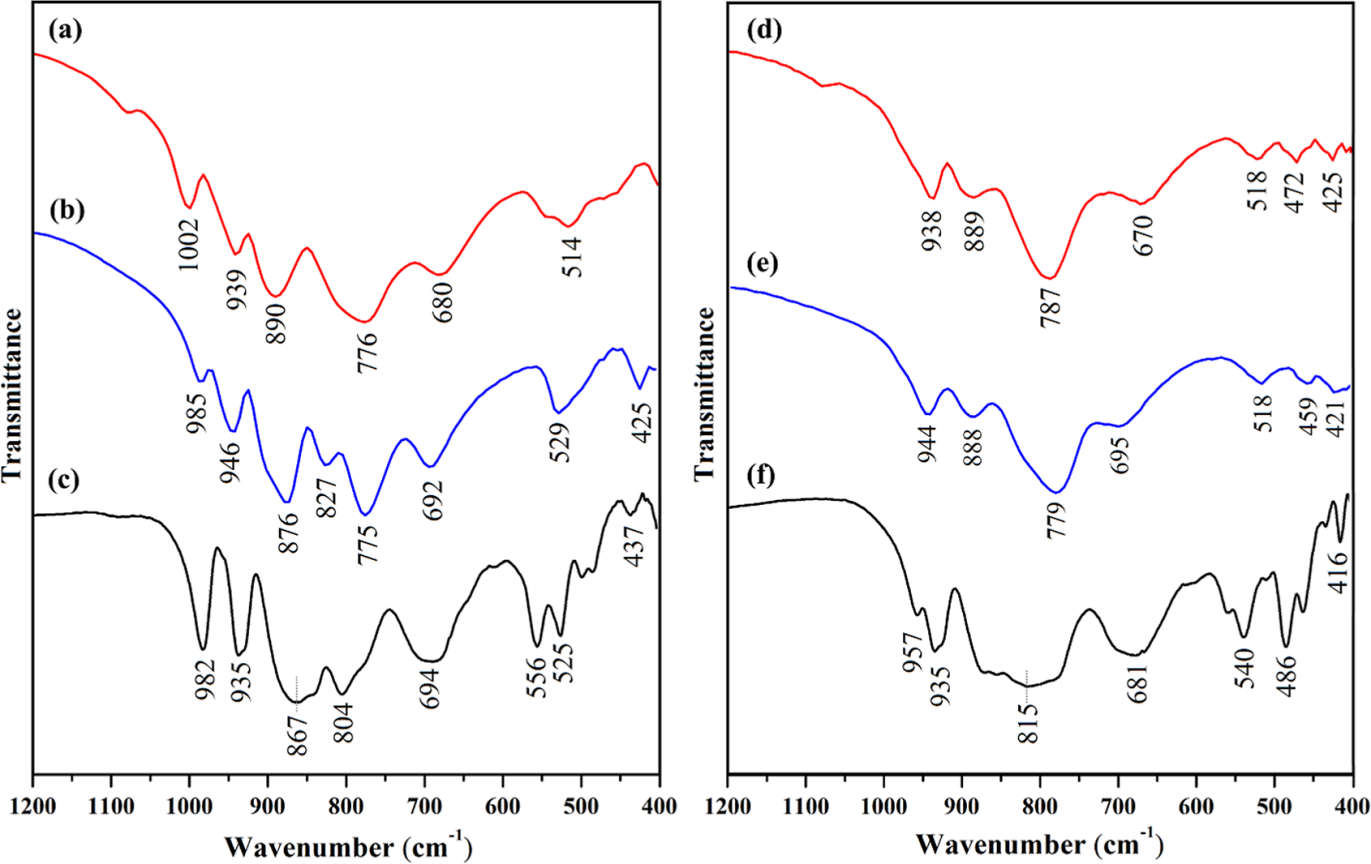
FT-IR spectra of (a) **RbNa–Th**_**3**_ **O**_**2**_ **Si**, (b) **RbNa–Th**_**3**_ **Si**, and (c) **Na–SiW**_**9**_, (d) **RbNa–Th**_**3**_ **O**_**2**_ **Ge**, (e) **RbNa–Th**_**3**_ **Ge**, and (f) **Na-GeW**_**9**_.

### Raman Spectroscopy

Peroxo-thorium species often do not exhibit a corresponding IR band but instead display a Raman band, indicative of a bridging-type peroxo group.^24^ In our case as well, solid-state Raman spectroscopy enables clear differentiation between the peroxo and nonperoxo compounds. As shown in Figure 6, the Raman spectra of the **Na–SiW**_**9**_ and **Na–GeW**_**9**_ Keggin precursors present a strong peak at around 934–963 cm^–1^ and 857–887 cm^–1^, corresponding to the typical asymmetric and symmetric stretching modes of the W=O_terminal_ and W–O–W bridging bonds in the Keggin skeleton.^23^ In Figure 6a, the spectrum of **RbNa–Th**_**3**_ **O**_**2**_ **Si** reveals a shift of these stretching modes to higher wavenumbers, with peaks observed at 954 and 887 cm^–1^, respectively. In peroxo–metal complexes, the Raman-active ν­(O–O) stretching vibrations are typically observed in the 870–825 cm^–1^ region.^9c–9e,25^ Consistent with this, the spectrum of polyanion **RbNa–Th**_**3**_ **O**_**2**_ **Si** exhibits two bands at 869 and 829 cm^–1^ due to the ν­(O–O) mode. Similarly, the same peroxo-mode can be observed for **RbNa–Th**_**3**_ **O**_**2**_ **Ge** at 864 and 826 cm^–1^, respectively. A band at approximately the same position, 859 cm^–1^, has been reported for Th­(O_2_)­(SO_4_)­(H_2_O)_2_.^21^ It is noteworthy that the Raman spectral bands particularly attributed to ν­(O–O) stretching modes decrease with increasing atomic number of the metals within a given group. It has been previously reported that discrete peroxo-uranyl­(VI) ions, such as [(UO_2_)_20_(O_2_)_28_(OH)_16_]^32–^ and [(UO_2_)_24_(O_2_)_36_(OH)_12_]^36–^, in the solid state exhibit a Raman band in the range 800–850 cm^–1^, respectively, assigned to the ν­(O–O) stretching modes.^26^ Similarly, in the case of polyanion [{(UO_2_)_4_(O_2_)_4_}_2_(P_8_W_48_O_184_)]^40–^, the peroxo bands have been observed at 855 and 827 cm^–1^, respectively.^25^ As expected, the characteristic bands of the O–O group are not present in the Raman spectra of the POM precursors and the nonperoxo analogues (see Figure 6), confirming the coordination of a peroxo group to the Th_3_ core in both polyanions **Th**_**3**_ **O**_**2**_ **Si** and **Th**_**3**_ **O**_**2**_ **Ge**, respectively.

**Figure 6:**
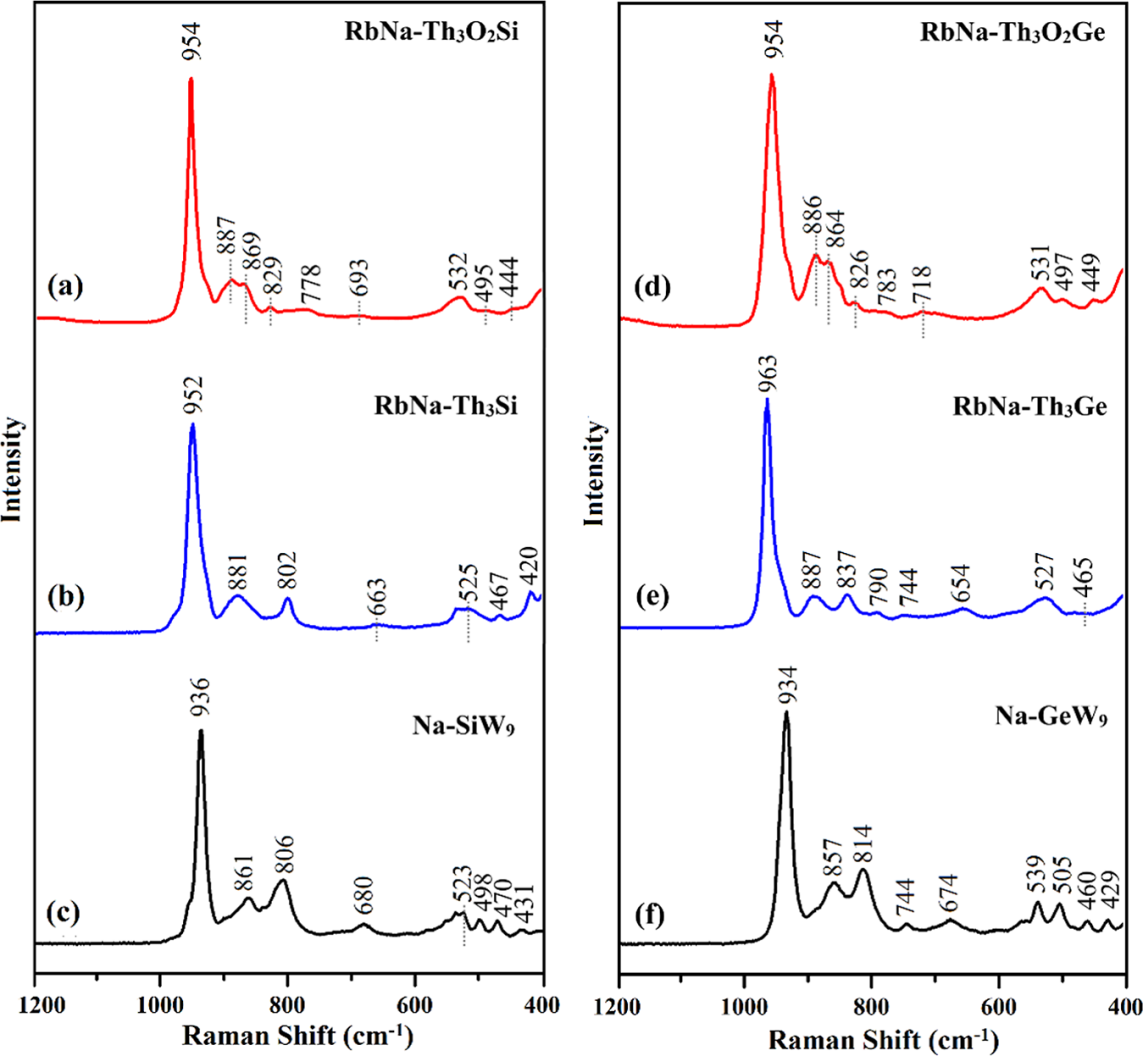
Solid-state Raman spectra of (a) **RbNa–Th**_**3**_ **O**_**2**_ **Si**, (b) **RbNa–Th**_**3**_ **Si**, (c) **Na–SiW**_**9**_, (d) **RbNa–Th**_**3**_ **O**_**2**_ **Ge**, (e), **RbNa–Th**_**3**_ **Ge**, and (f) **Na-GeW**_**9**_.

Figure 7 illustrates the solid-state Raman spectra of **RbNa–Th**_**3**_ **O**_**2**_ **Si** and **RbNa–Th**_**3**_ **O**_**2**_ **Ge** following controlled thermal treatment. The salts of the polyanions were gradually heated from 25 °C to the target temperature (60, 90, and 120 °C) at a rate of 5 °C/min under a continuous flow of N_2_ gas, and then cooled prior to spectral acquisition. All the spectra exhibit a strong band at approximately 954 cm^–1^, assigned to the W=O_terminal_ stretching vibrations. When comparing the spectra taken at room temperature, 60 °C, and 90 °C, the characteristic bands remain at nearly identical positions, highlighting the good thermal stability of the compounds. Notably, the ν­(O–O) modes in the range of 820–870 cm^–1^ can be detected even upon heating to 90 °C, further confirming the thermal robustness of the peroxo groups encapsulated within the polyanion framework. However, after thermal treatment at 120 °C, the bands in the 750–900 cm^–1^ region become broadened and the characteristic peroxo O–O vibrational bands are no longer clearly discernible. This suggests the possibility of partial decomposition of the peroxo groups under elevated thermal conditions. Additionally, a new weak signal at 803 cm^–1^ appears in the spectrum of **RbNa–Th**_**3**_ **O**_**2**_ **Si** after heating at 120 °C. The origin of this band is unclear but may arise from changes in the local coordination environment around the Th–O_peroxo_ bonds induced by thermal treatment.

**Figure 7:**
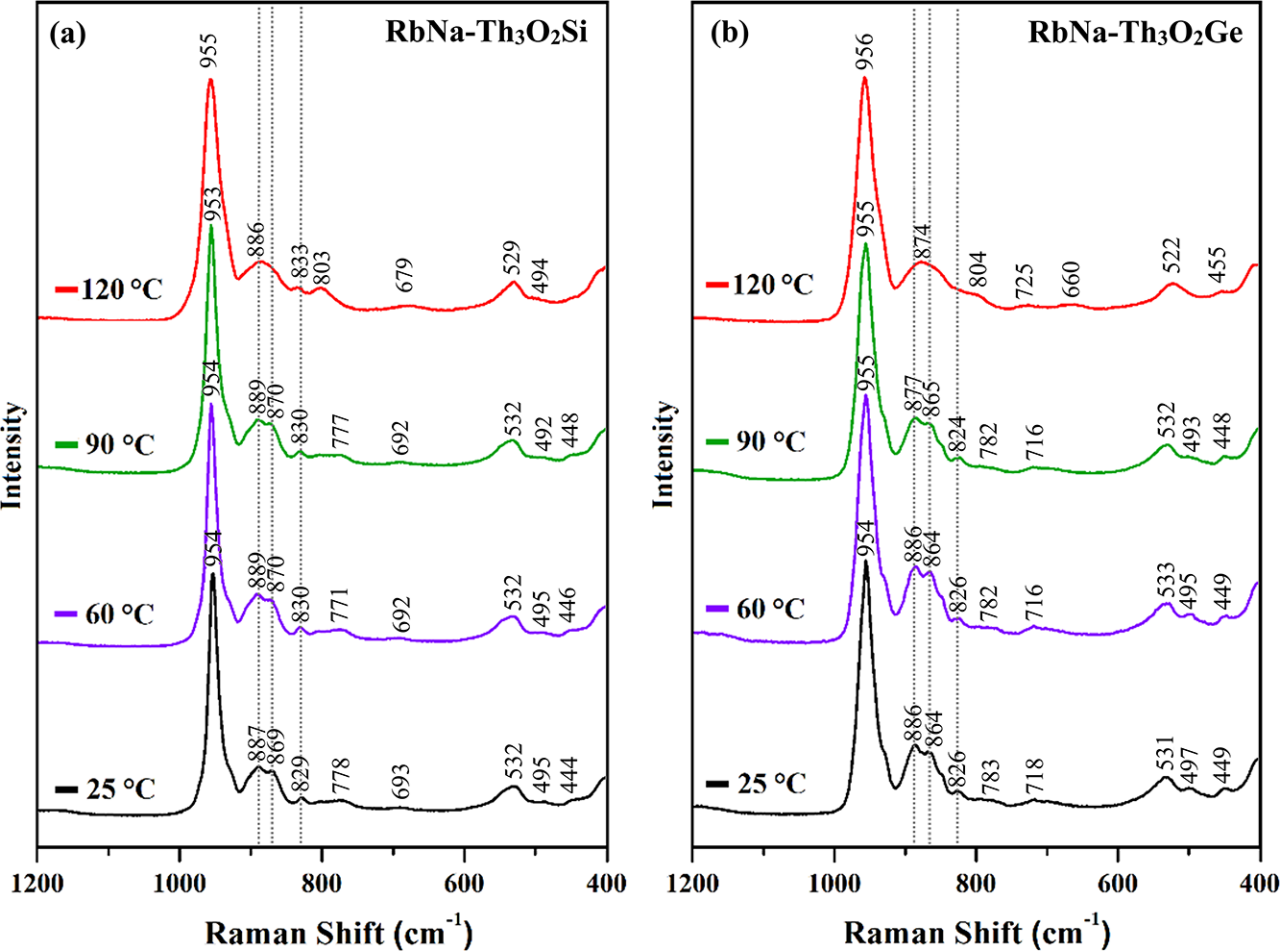
Solid-state Raman spectra of (a) **RbNa–Th**_**3**_ **O**_**2**_ **Si** and (b) **RbNa–Th**_**3**_ **O**_**2**_ **Ge** at room temperature and after thermal treatment at 60, 90, and 120 °C.

Raman spectra of aqueous solutions of all polyanions were recorded in water (with a concentration of 10 mM) at room temperature in the range of 400–1200 cm^–1^ using an exposure time of 16 s and a laser power of 100 mW. As expected, the solution-phase Raman spectra of all polyanions are very similar to their corresponding solid-state spectra, indicating the structural integrity of the polyanions in aqueous solution. However, slight shifts in the band positions are observed (Figure S8), which can be attributed to changes in the local environment of the polyanions upon dissolution (interaction with counter cations, water molecules etc.). Most importantly, the characteristic spectral bands corresponding to the ν­(O–O) stretching mode of the peroxo groups in the peroxo-thorium polyoxoanions **Th**_**3**_ **O**_**2**_ **X** can also be observed in the solution spectra. This suggests the structural stability of the peroxo group, which is well-stabilized by the polyanion framework also in solution.

### NMR Spectroscopy

The solution stability of the polyanions was further investigated using ^183^W NMR spectroscopy at room temperature in 1 M lithium acetate buffer (pH 4), to ensure a stable pH close to the synthetic conditions and to increase POM solubility in the presence lithium cations. Owing to the low solubility of the rubidium–sodium salts, the more soluble potassium–sodium salts were used instead to reach adequate conditions for ^183^W NMR analysis. As shown in Figure 8, all POMs display the expected two distinct peaks with a 2:1 intensity ratio in ^183^W NMR, corresponding to the 12 “belt” and 6 “cap” tungsten atoms. These results imply idealized *D*_*3h*_ symmetry in solution, different from the situation in the solid state (vide supra), probably due to rapid protonation and coordination dynamics within the central Th_3_ unit with respect to the NMR time scale.

**Figure 8:**
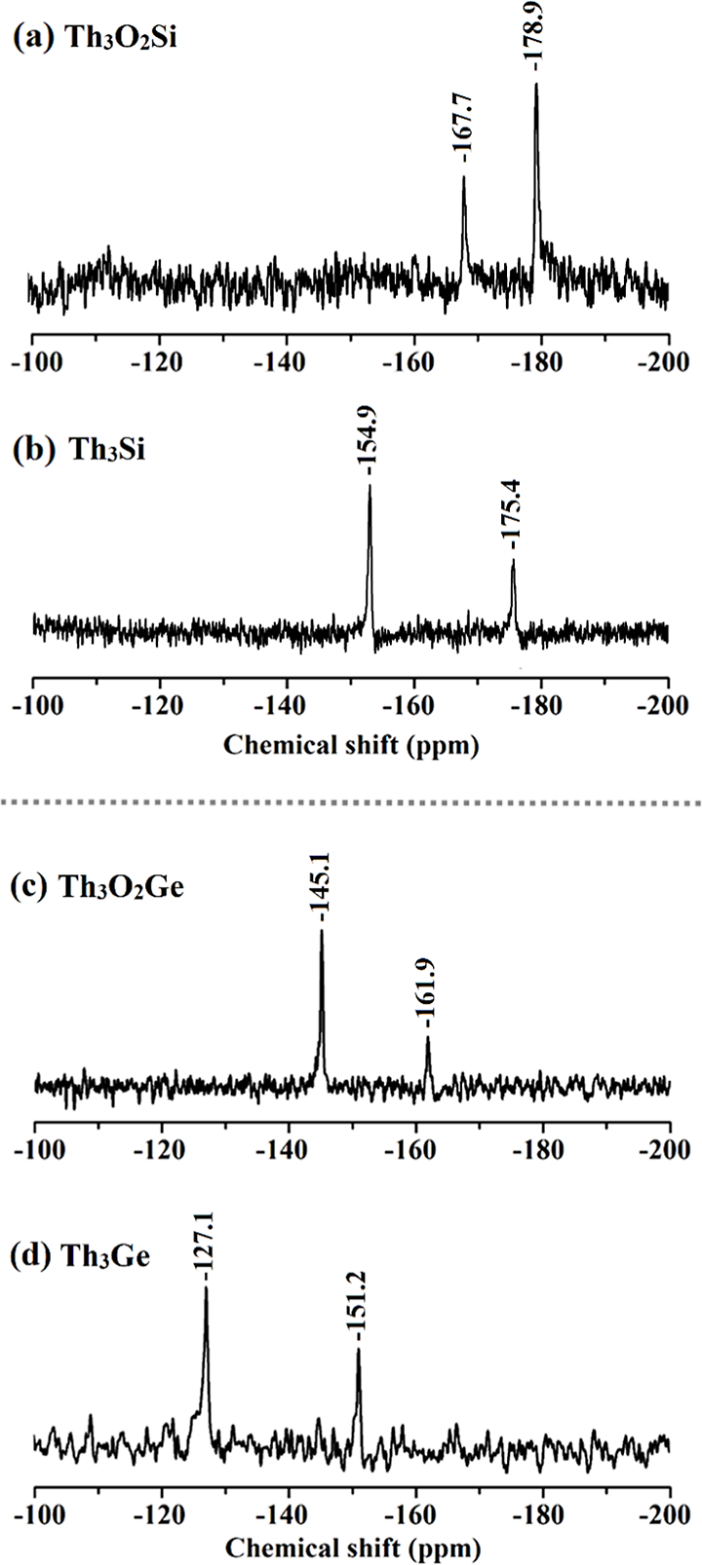
Room-temperature ^183^W NMR spectra of mixed potassium–sodium salts of (a) **Th**_**3**_ **O**_**2**_ **Si**, (b) **Th**_**3**_ **Si**, (c) **Th**_**3**_ **O**_**2**_ **Ge**, and (d) **Th**_**3**_ **Ge** dissolved in 1 M lithium acetate solution (pH 4).

It should be noted that the polyanions tend to recrystallize in the NMR tube after 2 days of data collection, reflecting a high stability (Table S5–S8). An upfield shift was noted for the ‘belt’ tungstens upon loss of peroxo in both analogues, from −178.9 to −154.9 for **Th**_**3**_ **O**_**2**_ **Si** and **Th**_**3**_ **Si** and from −145.1 to −127.1 for **Th**_**3**_ **O**_**2**_ **Ge** and **Th**_**3**_ **Ge**. The signals for the belt tungstens are more upfield than the ones for the caps for **Th**_**3**_ **Si**, **Th**_**3**_ **O**_**2**_ **Ge** and **Th**_**3**_ **Ge**, but not for **Th**_**3**_ **O**_**2**_ **Si** where the sequence is reversed. Computational analyses are probably needed to better understand this unexpected peculiarity. As shown in Figure S9, the ^29^Si NMR spectra of **Th**_**3**_ **O**_**2**_ **Si** and **Th**_**3**_ **Si** (dissolved in water) exhibit a single peak at −85.4 and −83.4 ppm, respectively, corresponding to the equivalent Si atoms within the {SiW_9_} subunits. The observed chemical shifts are in good agreement with previously reported values.^14,27^

### Electrochemistry

The electrochemical behavior of two peroxo–thorium­(IV)-containing heteropolytungstates, **Th**_**3**_ **O**_**2**_ **Si** and **Th**_**3**_ **O**_**2**_ **Ge**, together with their oxo analogues **Th**_**3**_ **Si** and **Th**_**3**_ **Ge**, was investigated in aqueous media between pH 1 and 6.5. No signal was observed for pH upper to pH 4. The electrochemical data has been reported in Table 2.

**Table 2:** Electrochemical Data of the Rubidium–Sodium Salts of **Th**_**3**_ **Si**, **Th**_**3**_ **O**_**2**_ **Si**, **Th**_**3**_ **Ge**, and **Th**_**3**_ **O**_**2**_ **Ge**.^a^

compounds	pH	W(VI)/W(V)
**Th**_**3**_ **Si**	pH = 1	–0.554
	pH = 2	–0.635
	pH = 2.5	–0.665
	pH = 3	–0.960^c^
		–1.140^c^
	pH = 4	–0.968
**Th**_**3**_ **O**_**2**_ **Si**	pH = 1	*–*0.337^e^
		–0.557
	pH = 2	–0.750
	pH = 2.5	–0.670^d^
		–1.068^c^
	pH = 3	–1.084^c^
	pH = 4	–0.881
		–1.007
**Th**_**3**_ **Ge**	pH = 1	–0.578
	pH = 2	–0.659
	pH = 2.5	–0.720
	pH = 3	–0.770
		–0.940
	pH = 4	–0.885
**Th**_**3**_ **O**_**2**_ **Ge**	pH = 2	–0.665
		–0.720
	pH = 3	–1.105^c^
	pH = 4	-^b^

a Potentials in V vs. SCE were obtained from cyclic voltammetry measured in aqueous solution at pH = 2 or pH = 4 in 0.5 M Na_2_SO_4_ + H_2_SO_4_. Working electrode: glassy carbon (GC) disk; auxiliary electrode: Pt wire, reference electrode: SCE. Scan rate: *v* = 0.1 V/s.

b Not observed.

c Not well-defined.

d Small signal.

e Peak potential measured using the convolution.

The electrochemical behavior of peroxo-Zr/Hf-containing undecatungstosilicates and -germanates, namely [M_2_(O_2_)_2_(α-XW_11_O_39_)_2_]^12–^ (abbreviated [(POM)_2_(O_2_)_2_]^12–^) where M = Zr^4+^, X = Si, Ge; or M = Hf^4+^, X = Si) have been already reported. The study indicated fast reductive release of the peroxo ligands upon reduction where the first reduction at pH 4.7 involved 8 electrons and 12H^+^: [(POM)_2_(O_2_)_2_]^12–^ + 8e^–^ + 12H^+^ → 2POMH_2_^4–^ + 4H_2_O.^28^

Figure 9a presents the cyclic voltammograms (CVs) of **Th**_**3**_ **Si** recorded at a scan rate of 100 mV/s within the potential range of −0.75 V to +1.20 V vs SCE at pH 2. The CV of **Th**_**3**_ **Si** displays a quasi-reversible reduction process at −0.635 V vs SCE, attributed to the W­(VI)/W­(V) couple corresponding to the two {SiW_9_} units.

**Figure 9:**
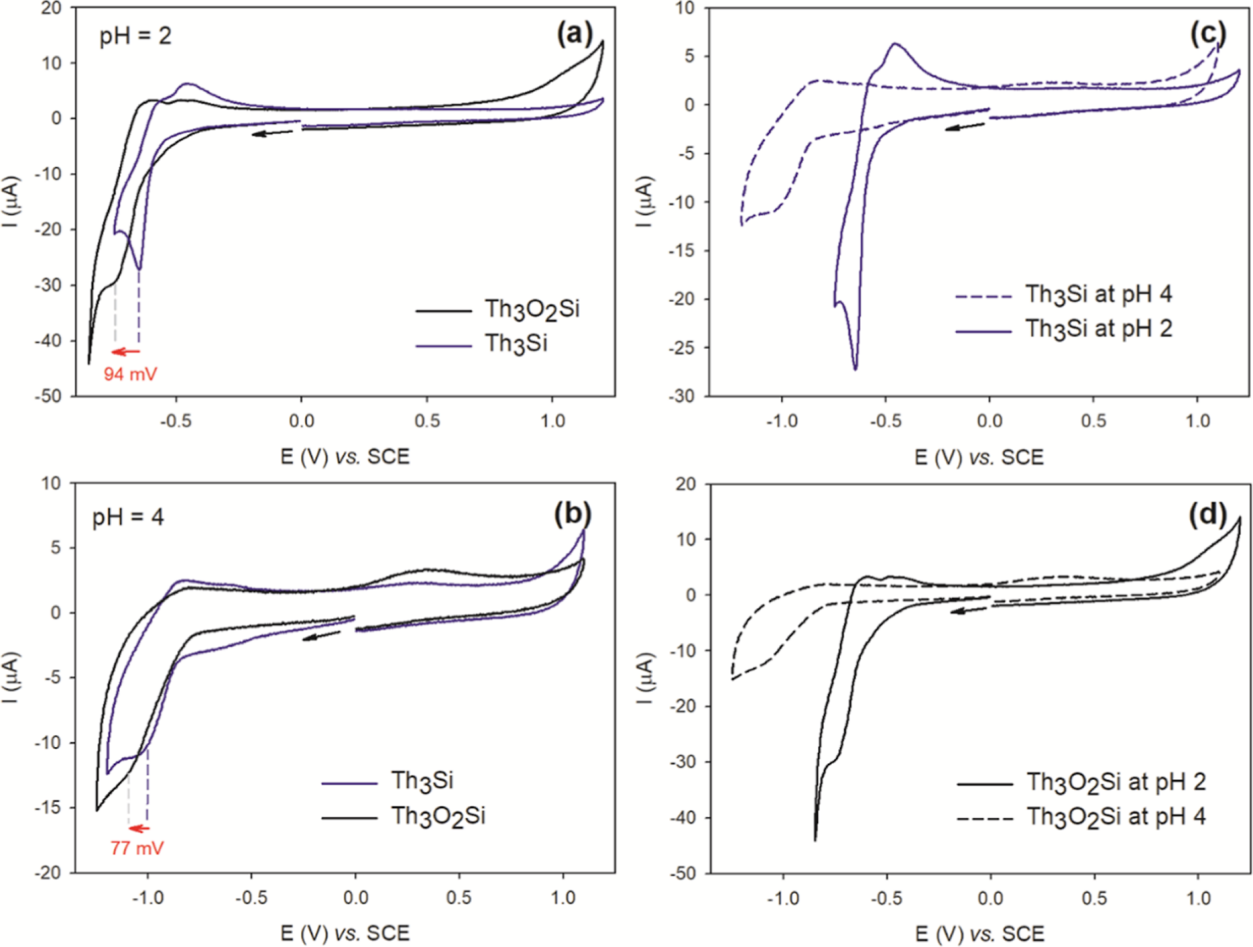
Cyclic voltammogram of **Th**_**3**_ **Si** and **Th**_**3**_ **O**_**2**_ **Si** (*c* = 0.5 mM) measured in aqueous solution at (a) pH 2 and (b) pH 4 containing 0.5 M Na_2_SO_4_ + H_2_SO_4_. (c) Comparison of the CVs of **Th**_**3**_ **Si** at pH 2 and pH 4. (d) Comparison of the CVs of **Th**_**3**_ **O**_**2**_ **Si** at pH 2 and pH 4. Working electrode: glassy carbon (GC) disk; auxiliary electrode: Pt wire, reference electrode: SCE. Scan rate: *v* = 0.1 V/s.

The peroxo–thorium­(IV) analogue **Th**_**3**_ **O**_**2**_ **Si** exhibits a comparable redox response, but the reduction occurs 94 mV more negatively at pH 2. Convolution analysis for **Th**_**3**_ **O**_**2**_ **Si** using the time semiderivative method further resolved this reduction into two closely spaced one-electron waves at −0.679 V and −0.729 V vs SCE separated by 50 mV consistent with weak electronic coupling between the {SiW_9_} units (Figure S10b). At pH 4, both compounds undergo reduction at more cathodic potentials, namely at −0.968 V for **Th**_**3**_ **Si**, and at −0.881 V and −1.007 V for **Th**_**3**_ **O**_**2**_ **Si** (Figure 9b and Table 2). At this pH, controlled potential electrolysis of the solution at the cathodic wave for **Th**_**3**_ **Si** showed the exchange of 1.95 electrons.

Single-crystal X-ray diffraction studies of the rubidium–sodium salts of the peroxo species [Th_3_(μ_3_-O_2_)­(OH)_2_(H_2_O)_3_(*A*-α-SiW_9_O_34_)_2_]^12–^ (**Th**_**3**_ **O**_**2**_ **Si**) and the oxo analogue [Th_3_(μ_3_-O)­(OH)_3_(H_2_O)­(*A*-α-SiW_9_O_34_)_2_]^13–^ (**Th**_**3**_ **Si**) revealed overall charges of −12 and −13, respectively. Based on charge considerations, the reduction of **Th**_**3**_ **Si** would be expected to occur at a more negative potential compared to **Th**_**3**_ **O**_**2**_ **Si**, in agreement with the electrostatic model generally applied to POMs, where an increase in total anionic charge enhances charge–charge repulsion and shifts the reduction potential cathodically.^29^

Contrary to this expectation, the experimental electrochemical data indicate the opposite trend. This discrepancy can be rationalized by protonation effects: at pH 2 and pH 4, protonation of the hydroxo bridges in both compounds likely alters their net charges, thereby modifying the reduction behavior.

The Pourbaix diagram has been tentatively plotted for **Th**_**3**_ **O**_**2**_ **Si** (Figure S21). Two slopes can be observed between pH 1 and 3 and between pH 3 and 4. Between pH 3 and 4, the slope measured is 111 mV/pH and 67 mV/pH, for the first and the second reduction, respectively. It suggests the reduction of the peroxo at the first reduction process which should involve 2e^–^ and 4H^+^ (with theoretical slope of 120 mV/pH if the process is rapid and reversible) which is expected to the reduction of the peroxo group, following by the reduction during the second step of the polyoxometalate subunits (W^VI/V^ couple) with at the same time protonation of the hydroxo groups. It must be noted that the shapes of CV for the peroxo-derivative may point to the intramolecular reduction of the peroxy unit by W^V^.

Comparable redox behavior was observed for **Th**_**3**_ **Ge** and **Th**_**3**_ **O**_**2**_ **Ge**, as illustrated in Figures S11 and S12. Furthermore, the Pourbaix diagram has been tentatively plotted for **Th**_**3**_ **Ge** (Figure S31). In the case of **Th**_**3**_ **Ge** a slope of 96 mV/pH (measured between pH 1 and 3) suggests the pick-up of 3 protons and 2 electrons during the reduction where the theoretical value assuming reversible and rapid process is expected to be 90 mV/pH. It suggests that the global reaction of the reduction mightbe

$${\left[{Th}_{3}\left(\mu_{3}‐O\right){\left(OH\right)}_{3}\left(H_{2}O\right){\left(A‐\alpha ‐{GeW}_{9}^{VI}O_{34}\right)}_{2}\right]}^{13-}+2e^{-}+3H^{+}\to {\left[{Th}_{3}\left(\mu_{3}‐O\right){\left(H_{2}O\right)}_{4}{\left(A‐\alpha ‐{GeW}_{9}^{VI}O_{34}\right)}_{2}\right]}^{12-}$$

As shown in Figure 10, the peak current of the reduction process for **Th**_**3**_ **O**_**2**_ **Si** increases linearly with the square root of the scan rate (ν^1/2^). This correlation demonstrates that the electron-transfer process is predominantly governed by diffusion. Similar results were observed for **Th**_**3**_ **Si**, **Th**_**3**_ **O**_**2**_ **Ge**, and **Th**_**3**_ **Ge** polyanions (Figures S13–S34). Moreover, cyclic voltammetry experiments confirm that **Th**_**3**_ **Si** remains electrochemically stable, as repeated reduction cycles do not lead to compound degradation.

**Figure 10:**
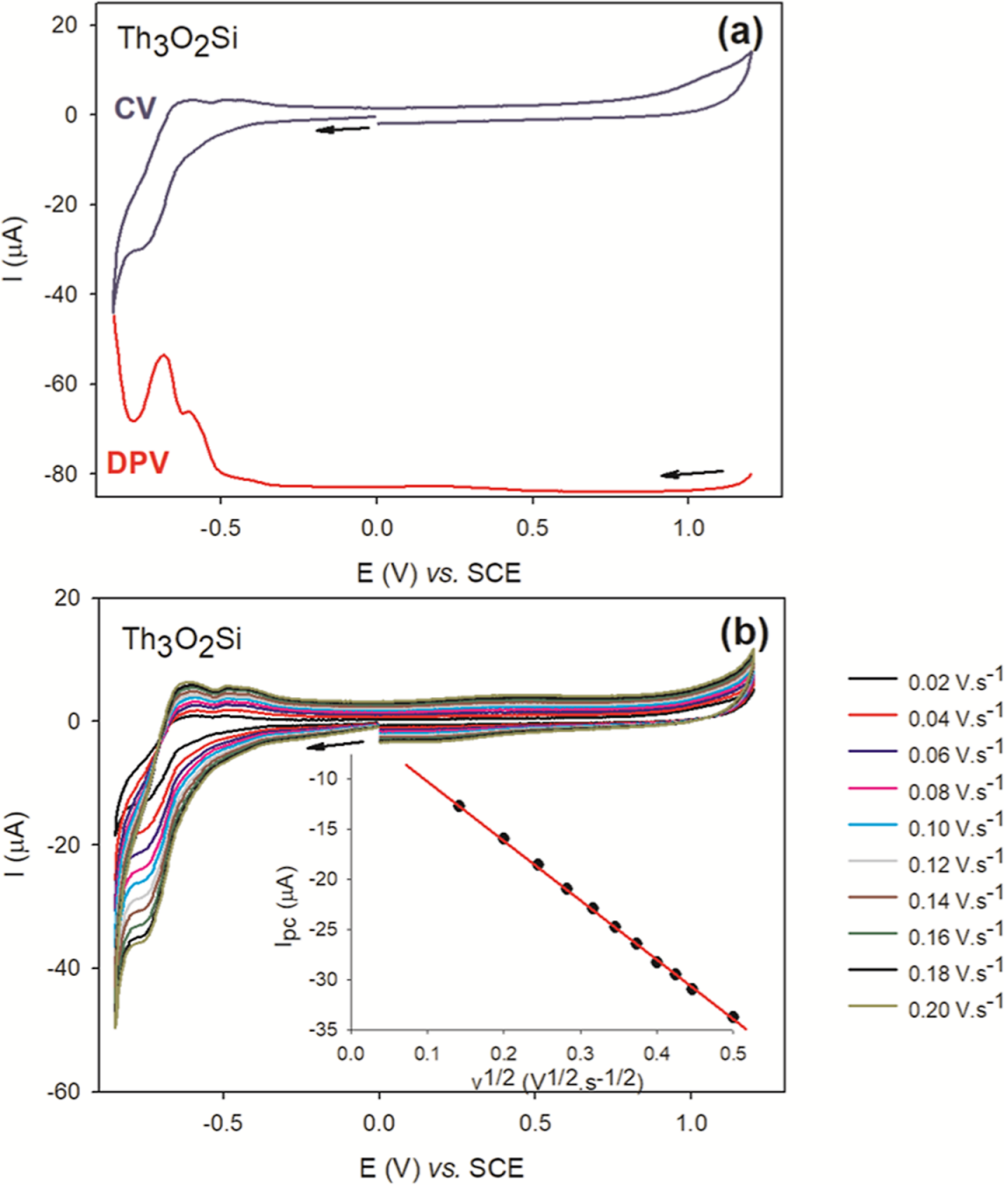
(a) Top: cyclic voltammogram (CV) of **Th**_**3**_ **O**_**2**_ **Si** (*c* = 0.5 mM) in aqueous solution at pH 2 containing 0.5 M Na_2_SO_4_ + H_2_SO_4_. Working electrode: glassy carbon (GC) disk; auxiliary electrode: Pt wire, reference electrode: SCE. Scan rate: *v* = 0.1 V/s; bottom: differential pulse voltammetry (DPV) of **Th**_**3**_ **O**_**2**_ **Si** (c = 0.5 mM). (b) Cyclic voltammogram of **Th**_**3**_ **O**_**2**_ **Si** measured at different scan rate from 0.02 V/s to 0.20 V/s. Inset: plot of *I*_pc_ vs *v* ^1/2^.

## Conclusions

We have successfully synthesized and structurally characterized the first two peroxo-thorium­(IV)-containing heteropolytungstates **Th**_**3**_ **O**_**2**_ **Si** and **Th**_**3**_ **O**_**2**_ **Ge**, respectively, in a simple one-pot reaction of thorium­(IV) ions and the trilacunary Keggin ions in aqueous medium in the presence of hydrogen peroxide. In the absence of hydrogen peroxide, but otherwise identical reaction conditions, the oxo-analogues **Th**_**3**_ **Ge** and **Th**_**3**_ **Si** were formed. Single-crystal XRD revealed that **Th**_**3**_ **O**_**2**_ **Si** and **Th**_**3**_ **O**_**2**_ **Ge** have a sandwich-type structure with a triangular arrangement of the three thorium ions and a central μ_3_-side-on peroxo group. ^183^W NMR and Raman spectroscopy demonstrated the stability of all four polyanions in solution. Notably, the two peroxo-POMs **Th**_**3**_ **O**_**2**_ **X** crystallized in the NMR tubes 2 days after the ^183^W NMR measurements had been performed, and single-crystal XRD as well as Raman spectroscopy confirmed the presence of the peroxo group. Moreover, solid-state Raman studies indicated stability of the peroxo group in **Th**_**3**_ **O**_**2**_ **X** even after heating to 90 °C. The electrochemical behavior of the peroxo species **Th**_**3**_ **O**_**2**_ **Si** and **Th**_**3**_ **O**_**2**_ **Ge** was compared with that of their oxo analogues **Th**_**3**_ **Ge** and **Th**_**3**_ **Si**. All four polyanions display a single reduction process; however, the reduction is shifted to more negative potentials for the peroxo-derivatives. While the oxo-analogues exhibit a single reduction wave, convolution analysis of the peroxo-species reveals splitting into two sequential one-electron processes, separated by 50–55 mV at pH 2, indicative of weak electronic coupling between the {XW_9_} subunits. These results provide valuable insights into the chemistry of peroxo-thorium complexes and advance the Frontier of actinide-based POM chemistry. Combined solid-state and solution studies reveal details of peroxo-thorium bonding, coordination behavior, and structural stability. Beyond their synthetic novelty, these compounds represent useful model systems for exploring *f*-block reactivity and may inspire future developments in actinide-based oxidation catalysis.

## Supplementary Material



## Notes

FT-IR, Raman and UV–vis spectra, thermograms, cyclic voltammograms, Pourbaix diagrams, bond valence sum calculations, and single-crystal XRD data (PDF)

## References

[1] a Pope, M. T. Heteropoly and Isopoly Oxometalates; Springer: Berlin, 1983.

[2] Hill C. L., Prosser-McCartha C. M. (1995). Homogeneous
Catalysis by Transition
Metal Oxygen Anion Clusters. Coord. Chem. Rev..

[3] Yuan C.-C., Wang S.-M., Chen W.-L., Liu L., Qin C., Su Z.-M., Wang E.-B. (2014). Polyoxometalate Supported Complexes
as Effective Electron-Transfer Mediators in Dye-Sensitized Solar Cells. Dalton Trans..

[4] Maksimchuk N. V., Evtushok V. Yu., Zalomaeva O. V., Maksimov G. M., Ivanchikova I. D., Chesalov Y. A., Eltsov I. V., Abramov P. A., Glazneva T. S., Yanshole V. V., Kholdeeva O. A., Errington R. J., Solé-Daura A., Poblet J. M., Carbó J. J. (2021). Activation
of H_2_O_2_ over Zr­(IV). Insights from Model Studies
on Zr-Monosubstituted Lindqvist Tungstates. ACS Catal..

[5] Beiles R. G., Rozmanova Z. E., Andreeva O. B. (1969). Preparation of Heteropolycompounds
with Peroxide Charactert. Russ. J. Inorg. Chem..

[6] Venturello C., Alneri E., Ricci M. (1983). A New Effective
Catalytic
System for Epoxidation of Olefins by Hydrogen Peroxide under Phase-Transfer
Conditions. J. Org. Chem..

[7] Droege M. W., Finke R. G. (1991). A Novel Triperoxyniobium-Containing
Polyoxoanion, SiW_9_(NbO_2_)_3_O_37_
^7‑^: Synthesis, Characterization, Catalytic Allylic
Epoxidations with
H_2_O_2_ and Preliminary Kinetic Studies. J. Mol. Catal..

[8] Judd D. A., Nettles J. H., Nevins N., Snyder J. P., Liotta D. C., Tang J., Ermolieff J., Schinazi R. F., Hill C. L. (2001). Polyoxometalate
HIV-1 Protease Inhibitors. A New Mode of Protease Inhibition. J. Am. Chem. Soc..

[9] Bassil B. S., Mal S. S., Dickman M. H., Kortz U., Oelrich H., Walder L. (2008). 6-Peroxo-6-Zirconium
Crown and Its
Hafnium Analogue Embedded in a Triangular Polyanion: [M_6_(O_2_)_6_(OH)_6_(γ-SiW_10_O_36_)_3_]^18‑^ (M = Zr, Hf). J. Am. Chem. Soc..

[10] Casellato U., Sitran S., Tamburini S., Vigato P. A., Graziani R. (1984). Lanthanide and Actinide Complexes
with Bidentate Ligands. Crystal Structure of Dimethylformamidetetrakis­(1-oxo-2-thiopyridinato)
Thorium­(IV). Inorg. Chim. Acta.

[11] Marcu G. H., Todorut I., Botar A. V. (1971). Studies
on Polyoxo and Polyperoxo-metalates: Part 7. Lanthano-and Thoriopolyoxotungstates
as Catalytic Oxidants with H_2_O_2_ and the X-ray
Crystal Structure of Na_8_[ThW_10_O_36_]·28H_2_O. Rev. Roum. Chim..

[12] Griffith W.
P., Morley-Smith N., Nogueira H. I. S., Shoair A. G. F., Suriaatmaja M., White A. J. P., Williams D. J. (2000). Studies on Polyoxo and Polyperoxo-metalates:
Part 7. Lanthano- and Thoriopolyoxotungstates as Catalytic Oxidants
with H_2_O_2_ and the X-ray Crystal Structure of
Na_8_[ThW_10_O_36_]·28H_2_O. J. Organomet. Chem..

[13] Ostuni A., Bachman R. E., Pope M. T. (2003). Multiple Diastereomers
of [M^n+^(*α*
_m_-P_2_W_17_O_61_)_2_]^(20‑n)‑^ (M = U^IV^, Th^IV^, Ce^III^; m = 1, 2). *Syn*- and *Anti*-Conformations of the Polytungstate
Ligands in α_1_α_1_, α_1_α_2_, and α_2_α_2_ Complexes. J. Clust. Sci..

[14] Duval S., Béghin S., Falaise C., Trivelli X., Rabu P., Loiseau T. (2015). Stabilization
of Tetravalent 4f (Ce), 5d (Hf), or 5f
(Th, U) Clusters by the [Α-SiW_9_O_34_]^10–^ Polyoxometalate. Inorg. Chem..

[15] Dufaye M., Duval S., Stoclet G., Loiseau T. (2019). Crystal Chemistry and
Saxs Studies of an Octahedral Polyoxoarsenotungstate Nanocluster Encapsulating
Four Unprecedented Thorium Arsenate Fragments ({Th_3_As_2_O_n_}– n = 25 or 26). Eur. J. Inorg. Chem..

[16] Bhattacharya S., Sarkar A., Zewdie T. A., George A. J., Dawoud S., Nisar T., Schürmann C. J., Wagner V., Ruhlmann L., Kortz U. (2025). Lanthanide and Actinide-Centered Polyoxo-Noble-Metalate-Based Metal-Organic
Frameworks. Chem. Asian J.

[17] Hervé G., Tézé A. (1977). Study of α- and β-Enneatungstosilicates
and -germanates. Inorg. Chem..

[18] Rigaku . Crysalispro Software System, version 1.171.38.41; Rigaku Oxford Diffraction, 2015

[19] Rigaku . ABSPACK, SCALE. “Empirical Absorption correction.”. In Crysalis Pro-software Package; Rigaku Oxford Diffraction, 2022.

[20] Sheldrick, G. M. SHELX, Program for Solution of Crystal Structures; University of Göttingen: Germany, 2015.

[21] Bonato L., Virot M., Dumas T., Mesbah A., Lecante P., Prieur D., Le Goff X., Hennig C., Dacheux N., Moisy P., Nikitenko S. I. (2019). Deciphering
the Crystal Structure
of a Scarce 1D Polymeric Thorium Peroxo Sulfate. Chem. Eur J..

[22] Brown I. D., Altermatt D. (1985). Bond-valence
Parameters Obtained from A Systematic
Analysis of the Inorganic Crystal Structure Database. Acta Crystallogr., Sect. B: Struct. Sci..

[23] Rocchiccioli-Deltcheff C., Fournier M., Franck R., Thouvenot R. (1983). Vibrational Investigations of Polyoxometalates.
2. Evidence for Anion-Anion Interactions in Molybdenum­(VI) and Tungsten­(VI)
Compounds Related to the Keggin Structure. Inorg.
Chem..

[24] Westland A.
D., Tarafder M. T. H. (1982). Novel
Peroxo
Complexes of Thorium Containing Organic Ligands. Inorg. Chem..

[25] Goura J., Sundar A., Bassil B. S., Ćirić-Marjanović G., Bajuk-Bogdanović D., Kortz U. (2020). Peroxouranyl-Containing
W_48_ Wheel: Synthesis, structure, and detailed Infrared
and Raman Spectroscopy study. Inorg. Chem..

[26] McGrail B. T., Sigmon G. E., Jouffret L. J., Andrews C. R., Burns P. C. (2014). Raman Spectroscopic
and ESI-MS Characterization of Uranyl Peroxide Cage Clusters. Inorg. Chem..

[27] Finke R. G., Rapko B., Weakley T. J. R. (1989). Polyoxoanions
derived from A-*β*-SiW_9_O_34_
^10‑^: Synthesis and Crystallographic and ^183^W NMR Characterization of Si_2_W_18_Zr_3_O_71_H_3_
^11‑^, Including Its Organic
Solvent Soluble Bu_4_N^+^ Salt. Inorg. Chem..

[28] Mal S. S., Nsouli N. H., Carraro M., Sartorel A., Scorrano G., Oelrich H., Walder L., Bonchio M., Kortz U. (2010). Peroxo-Zr/Hf-Containing
Undecatungstosilicates and -Germanates. Inorg.
Chem..

[29] Mbomekalle L. M., López X., Poblet J. M., Sécheresse F., Keita B., Nadjo L. (2010). Influence
of the Heteroatom Size
on the Redox Potentials of Selected Polyoxoanions. Inorg. Chem..

