# Supplementary material for: Peroxo-Thorium(IV)-Containing Heteropolytungstates and Their Oxo-Analogues: Synthesis, Structure and Solution Studies
Source: Inorg Chem. 2026 Jan 8;65(2):1065–77. doi: 10.1021/acs.inorgchem.5c04382 (PMC12820932; doi:10.1021/acs.inorgchem.5c04382)
Supplement: Supplementary file 1 [file ic5c04382_si_001.pdf]

Associated Content

Supporting Information

Peroxo-Thorium(IV)-Containing Heteropolytungstates and Their Oxo-Analogues: Synthesis, Structure and Solution Studies

Sahar Khandan,[a] Bassem S. Bassil,[a] Anupam Sarkar,[a] Ayush Kant Ranga,[a] Arnulf Materny,[a] Samer Dawoud,[b] Laurent Ruhlmann,[b] and Ulrich Kortz[a]*

[a] School of Science, Constructor University, Campus Ring 1, 28759 Bremen, Germany

[b] UMR CNRS 7177, Laboratoire d'Electrochimie et de Chimie Physique du Corps Solide, Institut de Chimie, Université de Strasbourg, 67081 Strasbourg, France

E-mail address: ukortz@constructor.university

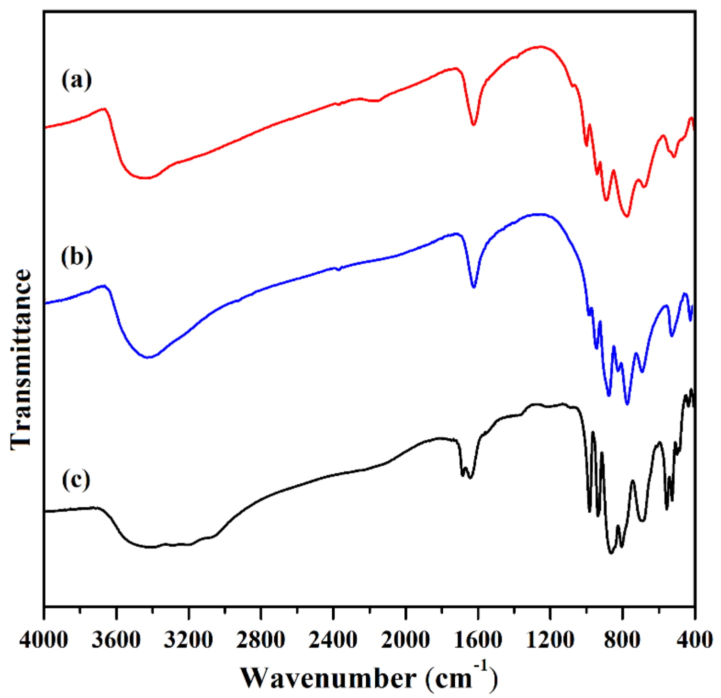

Figure S1. FT-IR spectra of (a) **RbNa-Th_3O_2Si**, (b) **RbNa-Th_3Si**, and (c) **Na-SiW_9** POM precursor.

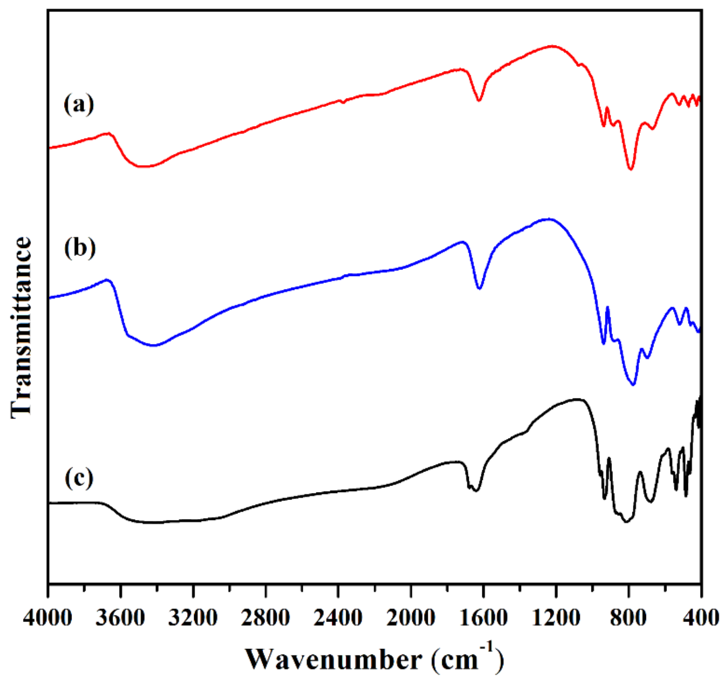

Figure S2. FT-IR spectra of (a) **RbNa-Th_3O_2Ge**, (b) **RbNa-Th_3Ge**, and (c) **Na-GeW_9** POM precursor.

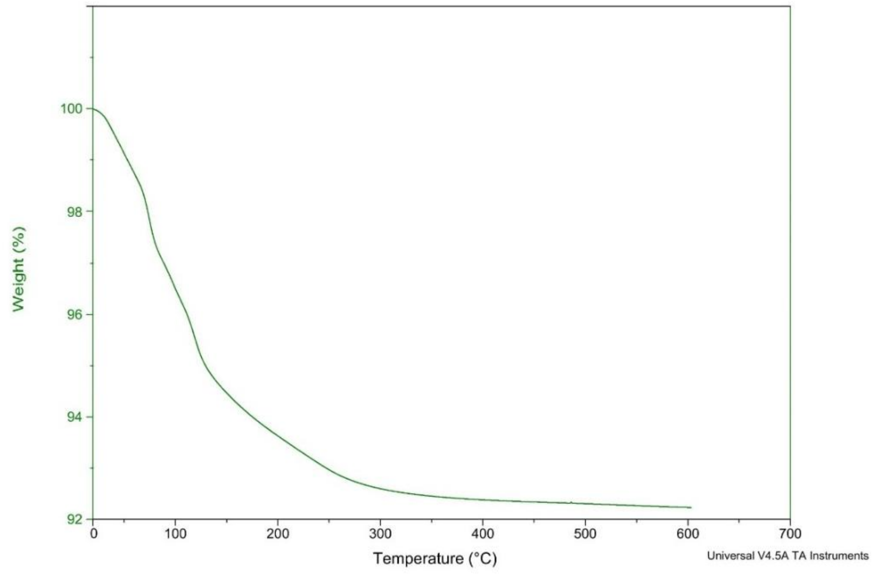

Figure S3. Thermogram of **RbNa-Th$_3$O$_2$Si** from room temperature to 600 ºC under N_2.

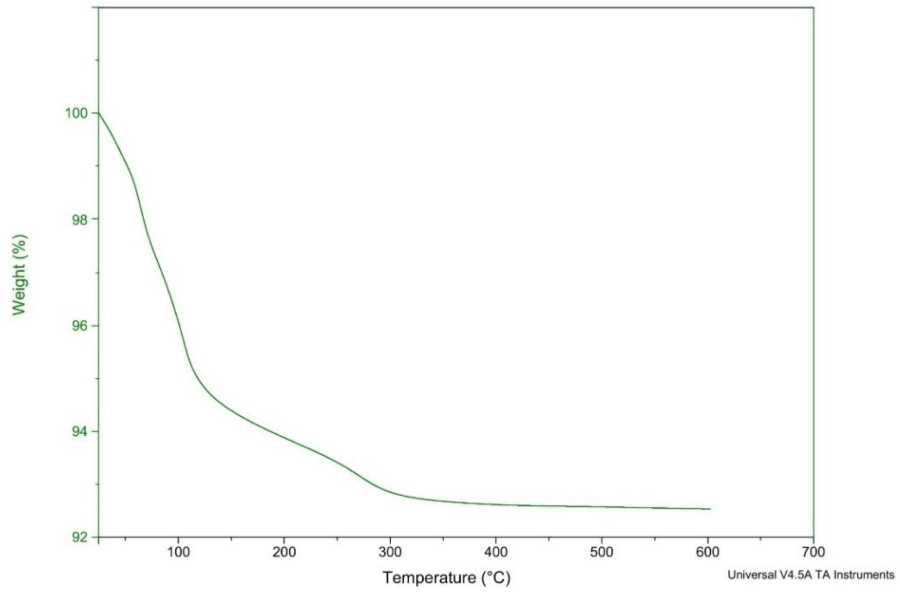

Figure S4. Thermogram of **RbNa-Th$_3$O$_2$Ge** from room temperature to 600 ºC under N_2.

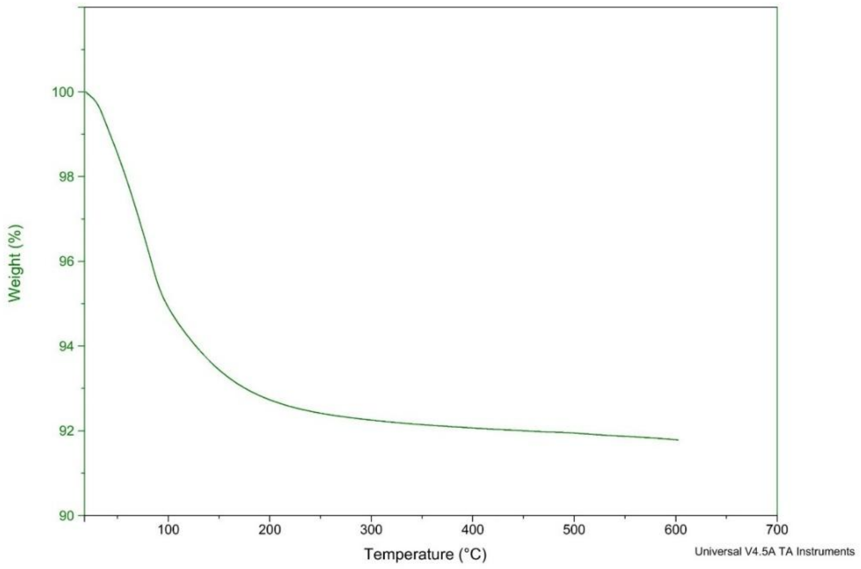

Figure S5. Thermogram of **RbNa-Th_3Si** from room temperature to 600 ºC under N_2.

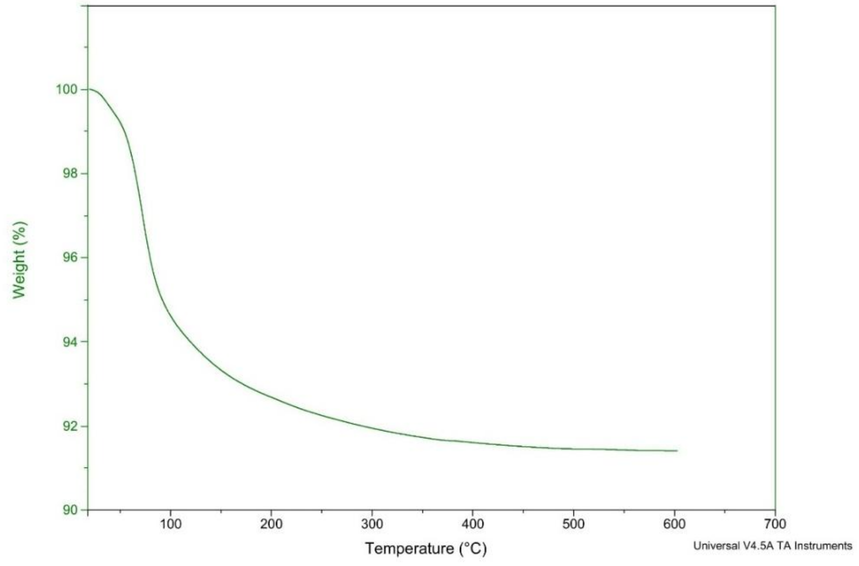

Figure S6. Thermogram of **RbNa-Th_3Ge** from room temperature to 600 ºC under N_2.

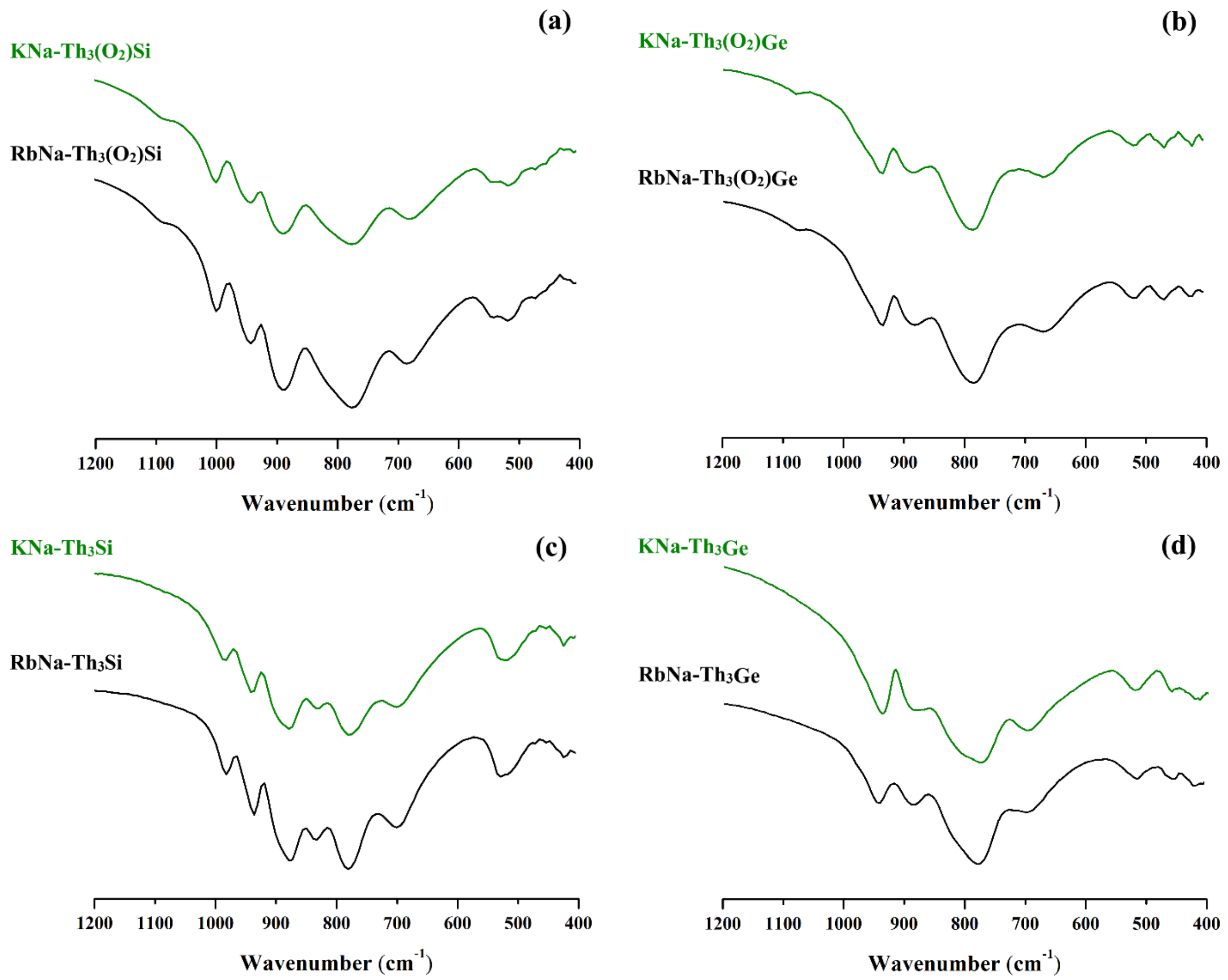

Figure S7. Comparison of the FT-IR spectra of the potassium-sodium (green) and rubidium-sodium salts (black) of the synthesized polyanions (a) **Th_3O_2Si**, (b) **Th_3O_2Ge**, (c) **Th_3Si**, and (d) **Th_3Ge** recorded in the range of 400-1200 cm^{-1}.

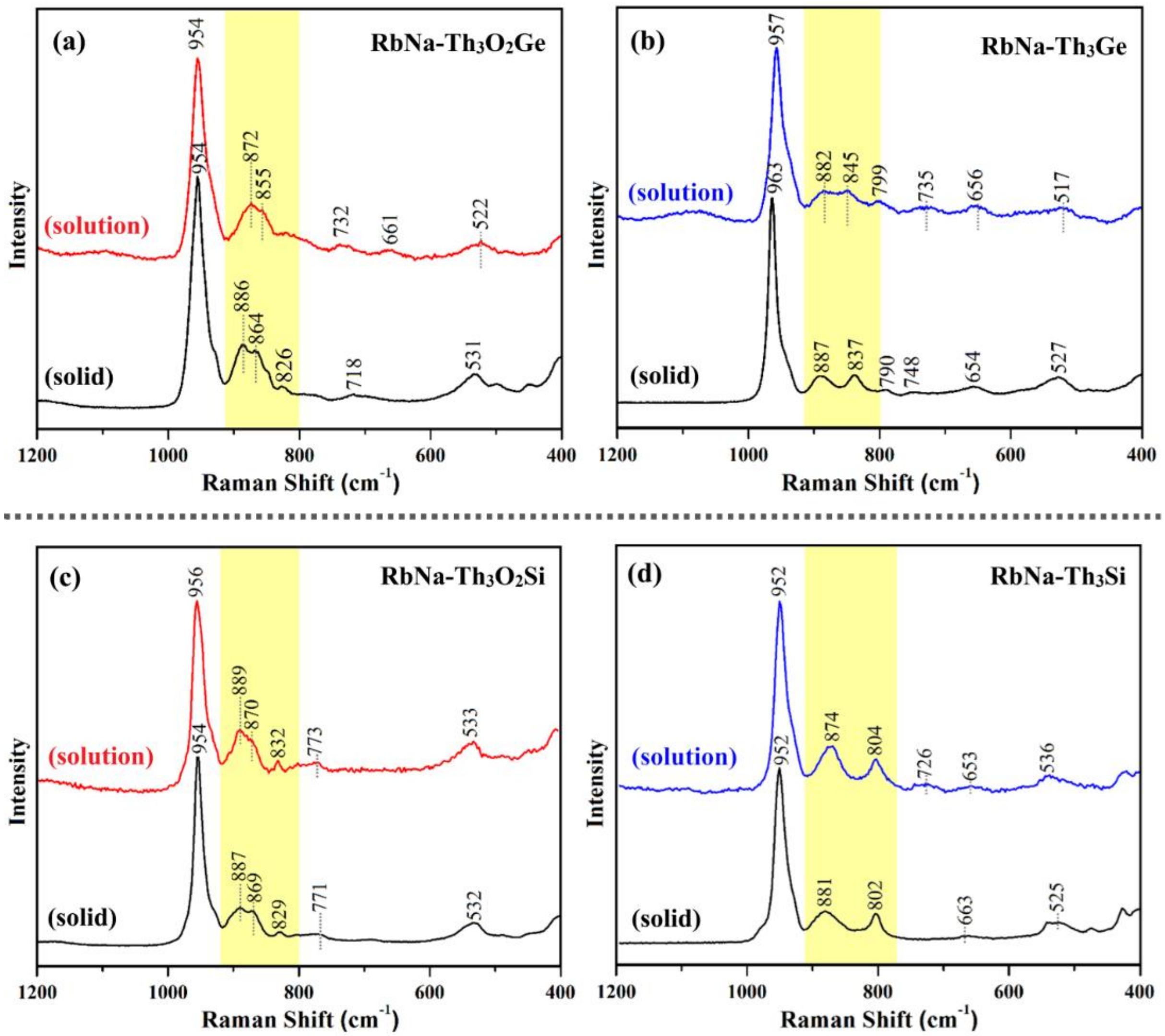

Figure S8. Raman spectra of (a) **RbNa-Th$_3$O$_2$Ge**, (b) **RbNa-Th$_3$Ge**, (c) **RbNa-Th$_3$O$_2$Si**, and (d) **RbNa-Th$_3$Si** in both solid-state and aqueous solution.

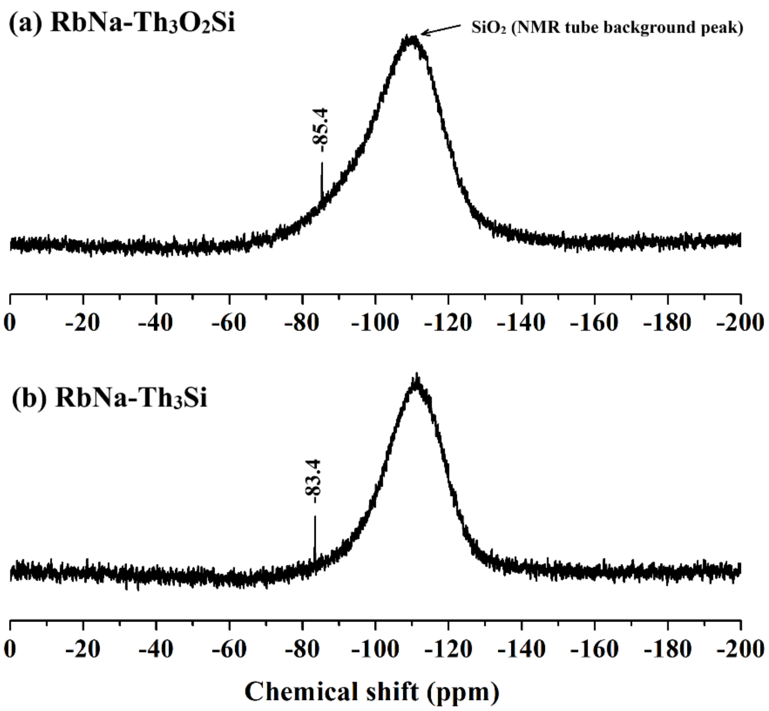

Figure S9. Room-temperature ^{29}Si NMR spectra of mixed rubidium-sodium salts of (a) **Th_3O_2Si** and (b) **Th_3Si** dissolved in water/D_2O.

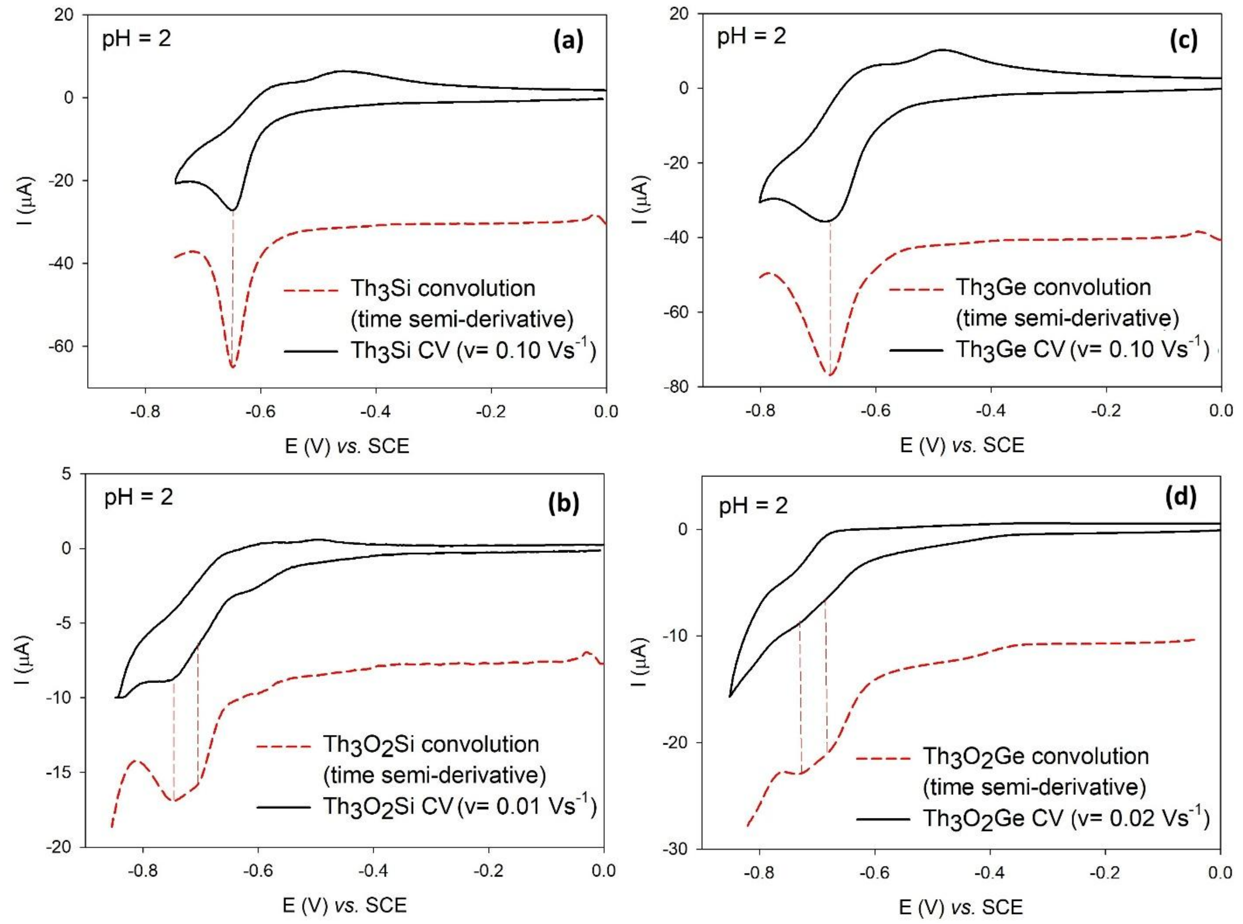

Figure S10. Cyclic voltammogram and the convolution (time semi-derivative) of (a) **Th_3Si**, (b) **Th_3O_2Si**, (c) **Th_3Ge**, and (d) **Th_3O_2Ge** (c = 0.5 mM) measured in aqueous solution at pH 2 in 0.5 M $Na_2SO_4 + H_2SO_4$. Working electrode: glassy carbon (GC) disk; auxiliary electrode: Pt wire, reference electrode: SCE. Scan rate: v = 0.01-0.10 V/s.

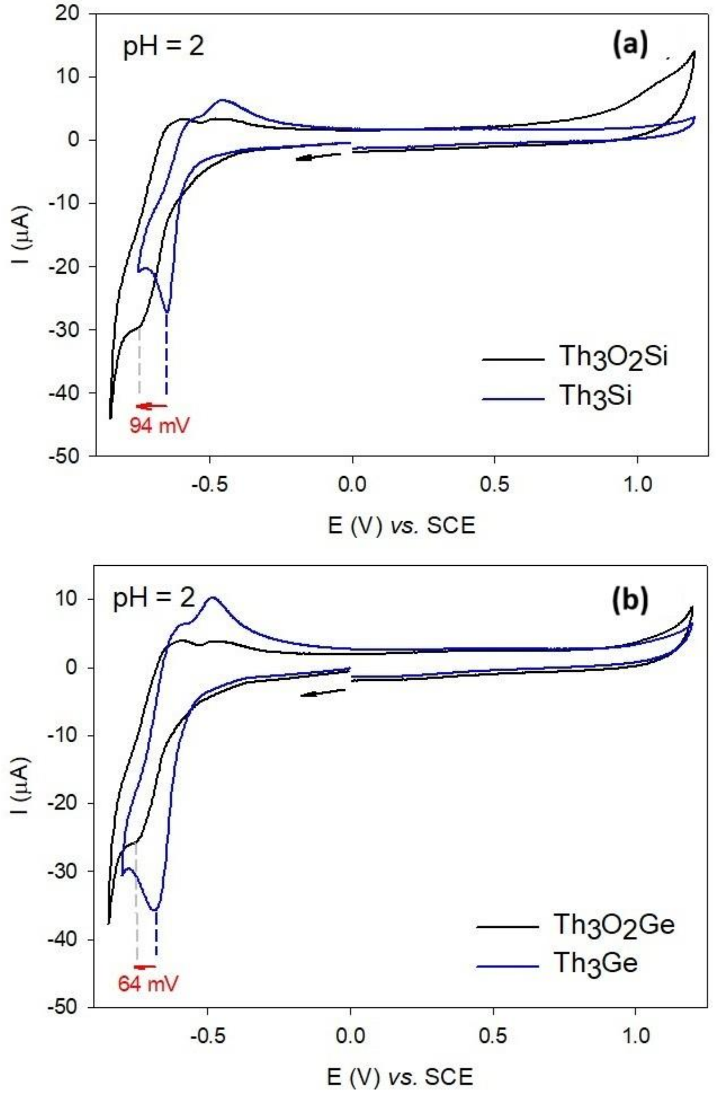

Figure S11. (a) Cyclic voltammogram of **Th_3Si** compared to **Th_3O_2Si** (c = 0.5 mM) measured in aqueous solution at pH 2 containing 0.5 M Na_2SO_4 + H_2SO_4. (b) cyclic voltammogram of **Th_3Ge** compared to **Th_3O_2Ge** measured in the same conditions. Working electrode: glassy carbon (GC) disk; auxiliary electrode: Pt wire, reference electrode: SCE. Scan rate: v = 0.1 V/s.

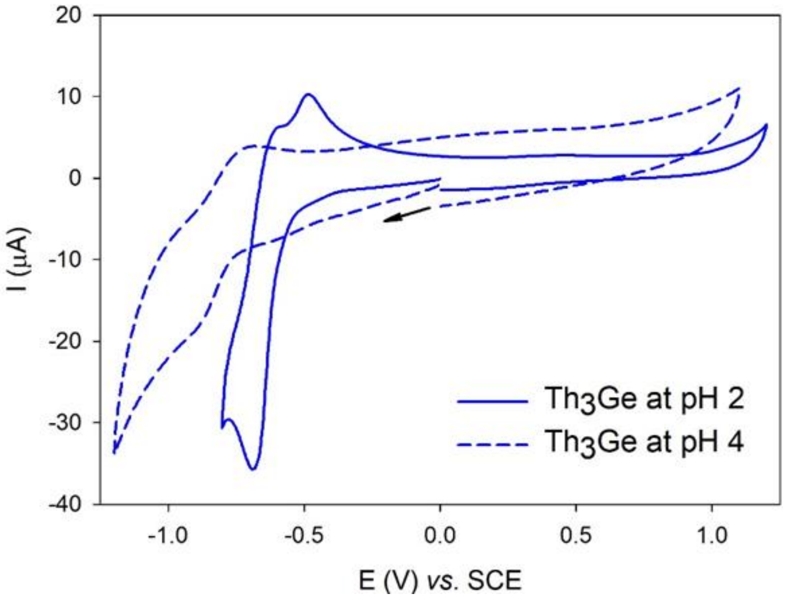

Figure S12. Cyclic voltammogram of **Th_3Ge** (c = 0.5 mM) measured in aqueous solution at pH 2 and pH 4 containing 0.5 M Na_2SO_4 + H_2SO_4. Working electrode: glassy carbon (GC) disk; auxiliary electrode: Pt wire, reference electrode: SCE. Scan rate: v = 0.1 V/s.

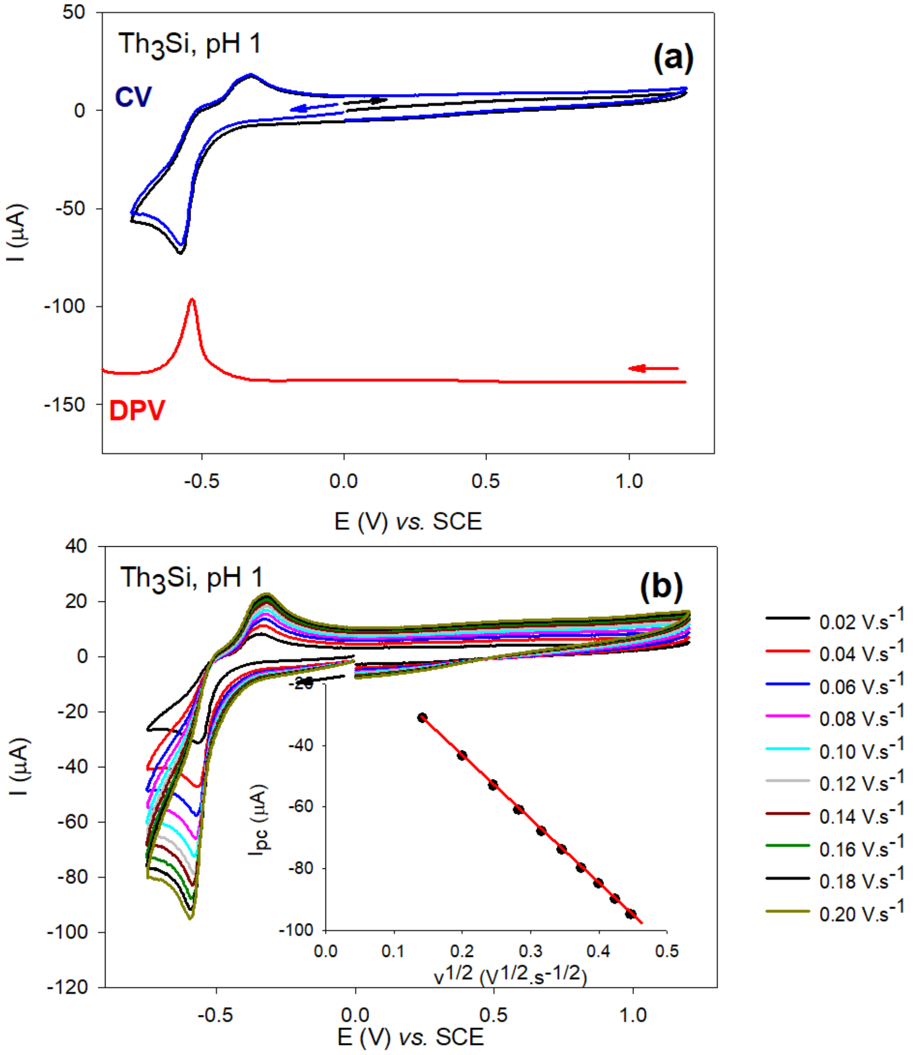

Figure S13. (a) top: cyclic voltammogram (CV) of **Th₃Si** (c = 0.5 mM) in aqueous solution at pH 1 containing 0.5 M Na_2SO_4 + H_2SO_4. Working electrode: glassy carbon (GC) disk; auxiliary electrode: Pt wire, reference electrode: SCE. Scan rate: v = 0.1 V/s; bottom: differential pulse voltammetry (DPV) of **Th_3Si** (c = 0.5 mM). (b) cyclic voltammogram of **Th_3Si** measured at different scan rate from 0.02 V/s to 0.20 V/s. Inset: Plot of I_{pc} *vs.* $v^{1/2}$.

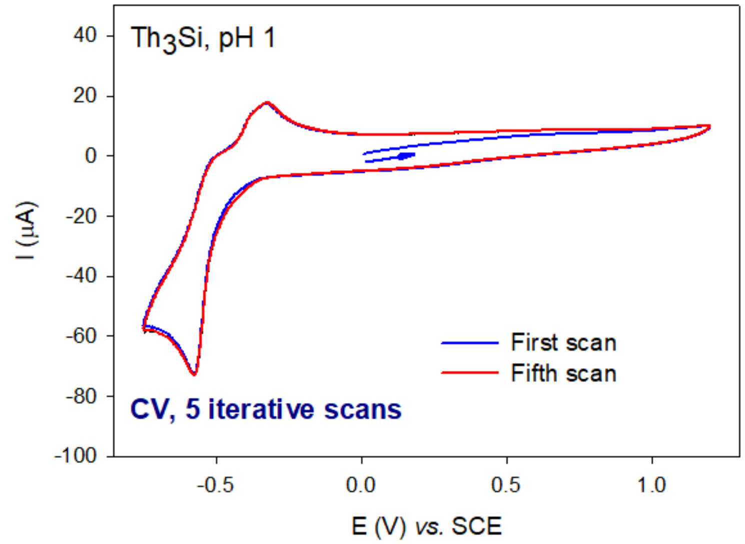

Figure S14. Cyclic voltammogram (CV) of **Th_3Si** (c = 0.5 mM) during 5 iterative cycles in aqueous solution at pH 1 containing 0.5 M Na_2SO_4 + H_2SO_4. Working electrode: glassy carbon (GC) disk; auxiliary electrode: Pt wire, reference electrode: SCE. Scan rate: v = 0.1 V/s.

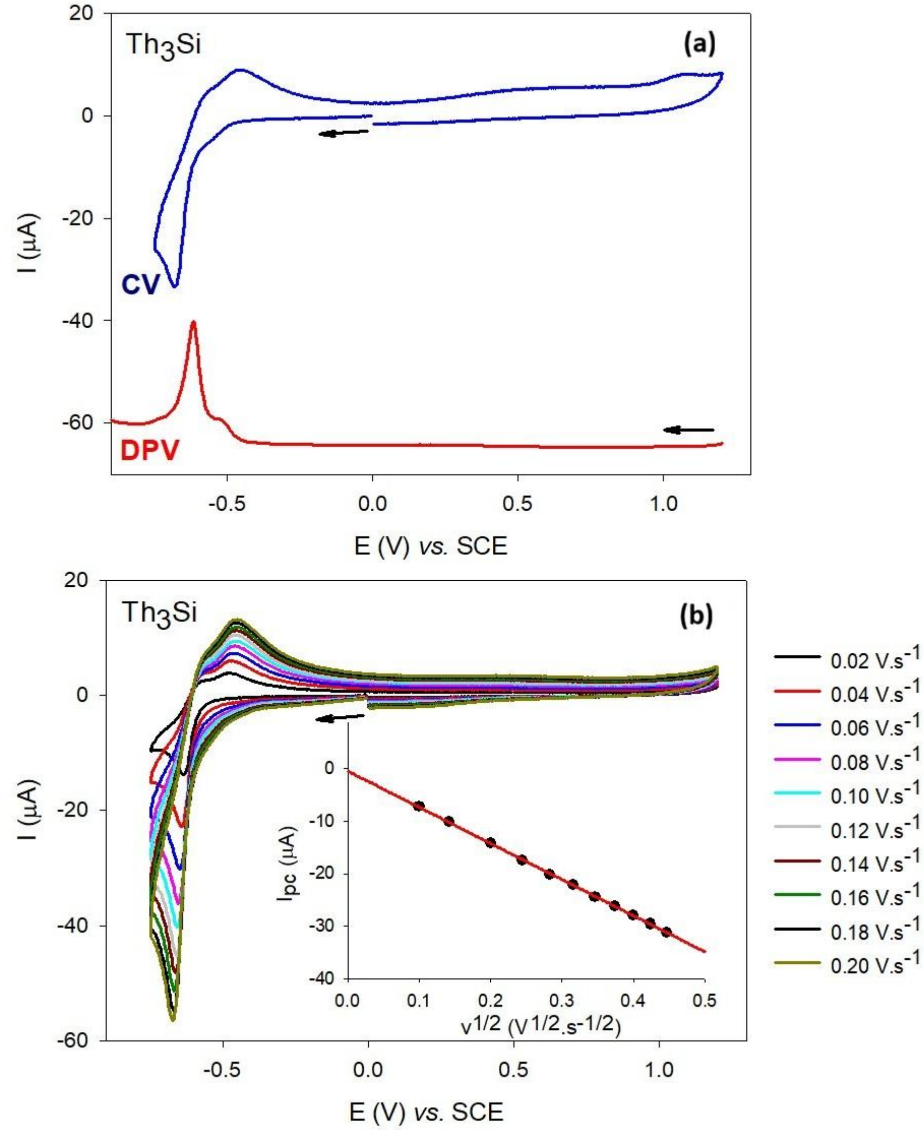

Figure S15. (a) top: cyclic voltammogram (CV) of **Th_3Si** (c = 0.5 mM) in aqueous solution at pH 2 containing 0.5 M Na_2SO_4 + H_2SO_4. Working electrode: glassy carbon (GC) disk; auxiliary electrode: Pt wire, reference electrode: SCE. Scan rate: v = 0.1 V/s; bottom: differential pulse voltammetry (DPV) of **Th_3Si** (c = 0.5 mM). (b) cyclic voltammogram of **Th_3Si** measured at different scan rate from 0.02 V/s to 0.20 V/s. Inset: Plot of I_{pc} *vs.* $v^{1/2}$.

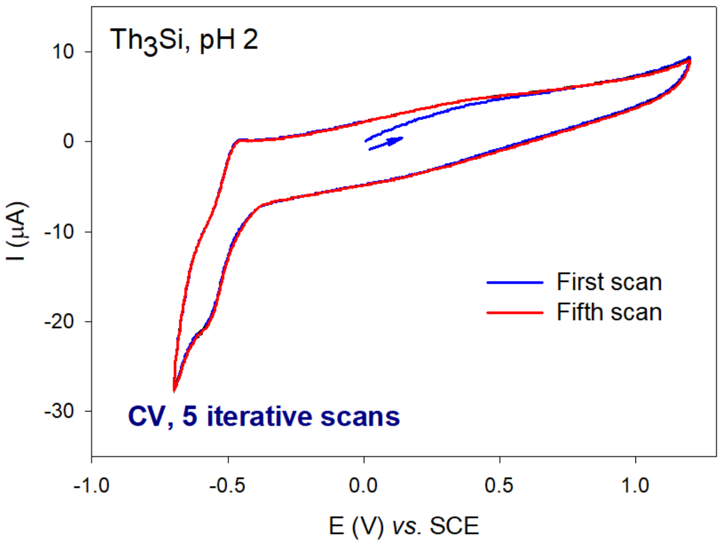

Figure S16. Cyclic voltammogram (CV) of **Th_3Si** (c = 0.5 mM) during 5 iterative cycles in aqueous solution at pH 2 containing 0.5 M Na_2SO_4 + H_2SO_4. Working electrode: glassy carbon (GC) disk; auxiliary electrode: Pt wire, reference electrode: SCE. Scan rate: v = 0.1 V/s.

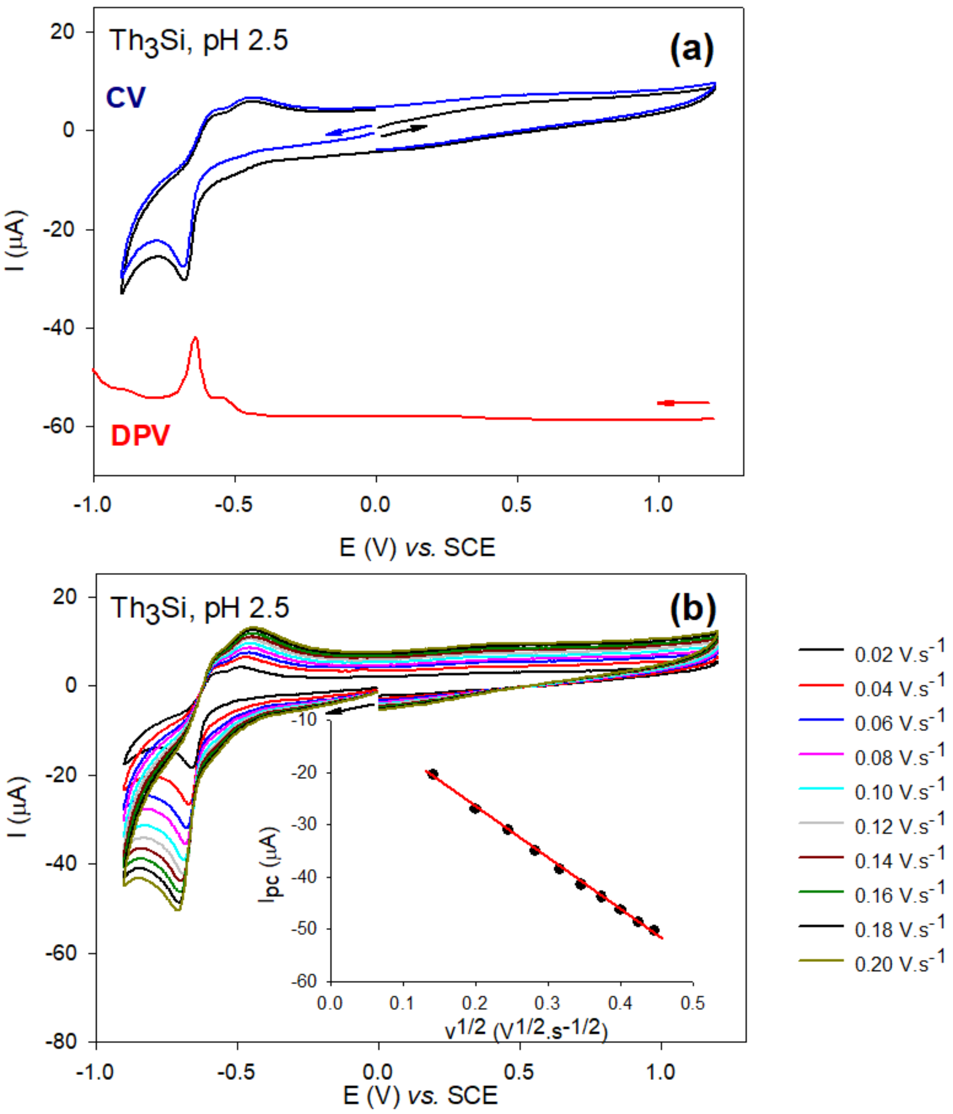

Figure S17. (a) top: cyclic voltammogram (CV) of **Th_3Si** (c = 0.5 mM) in aqueous solution at pH 2.5 containing 0.5 M Na_2SO_4 + H_2SO_4. Working electrode: glassy carbon (GC) disk; auxiliary electrode: Pt wire, reference electrode: SCE. Scan rate: v = 0.1 V/s; bottom: differential pulse voltammetry (DPV) of **Th_3Si** (c = 0.5 mM). (b) cyclic voltammogram of **Th_3Si** measured at different scan rate from 0.02 V/s to 0.20 V/s. Inset: Plot of I_{pc} *vs.* $v^{1/2}$.

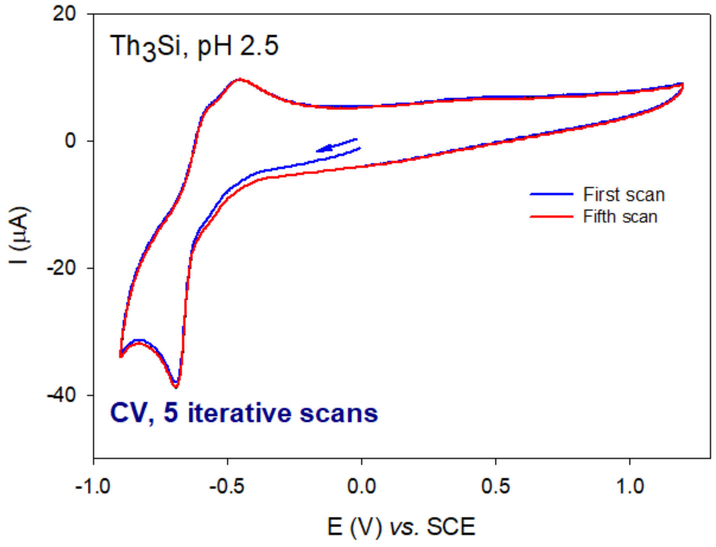

Figure S18. Cyclic voltammogram (CV) of **Th_3Si** (c = 0.5 mM) during 5 iterative cycles in aqueous solution at pH 2.5 containing 0.5 M Na_2SO_4 + H_2SO_4. Working electrode: glassy carbon (GC) disk; auxiliary electrode: Pt wire, reference electrode: SCE. Scan rate: v = 0.1 V/s.

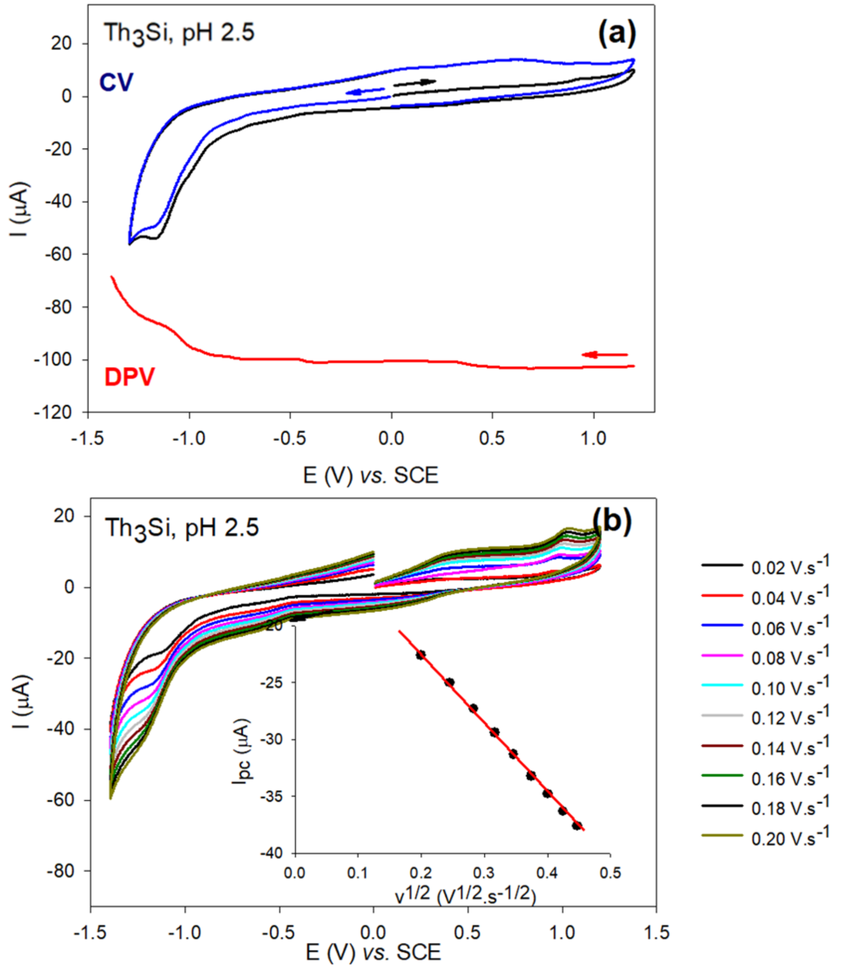

Figure S19. (a) top: cyclic voltammogram (CV) of **Th3Si** (c = 0.5 mM) in aqueous solution at pH 2.5 containing 0.5 M Na_2SO_4 + H_2SO_4. Working electrode: glassy carbon (GC) disk; auxiliary electrode: Pt wire, reference electrode: SCE. Scan rate: v = 0.1 V/s; bottom: differential pulse voltammetry (DPV) of **Th3Si** (c = 0.5 mM). (b) cyclic voltammogram of **Th3Si** measured at different scan rate from 0.02 V/s to 0.20 V/s. Inset: Plot of I_{pc} *vs.* $v^{1/2}$.

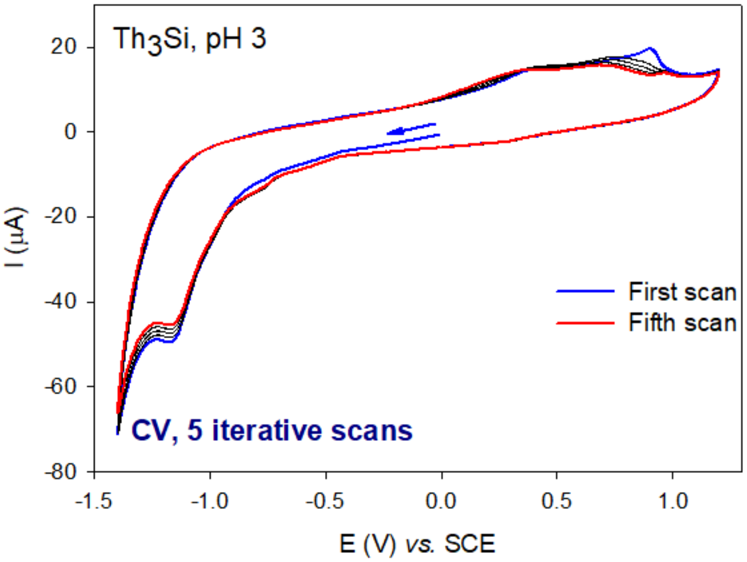

Figure S20. Cyclic voltammogram (CV) of **Th_3Si** (c = 0.5 mM) during 5 iterative cycles in aqueous solution at pH 3 containing 0.5 M Na_2SO_4 + H_2SO_4. Working electrode: glassy carbon (GC) disk; auxiliary electrode: Pt wire, reference electrode: SCE. Scan rate: v = 0.1 V/s.

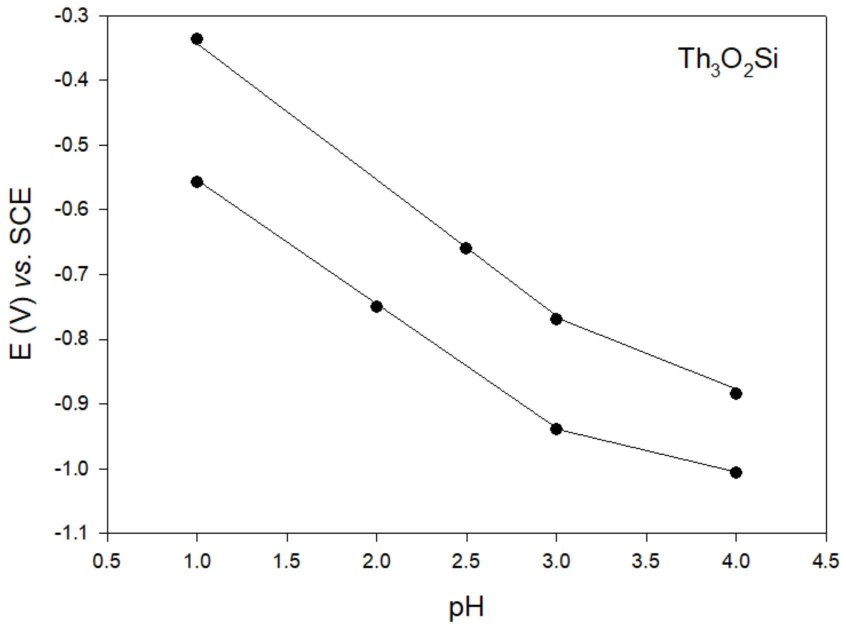

Figure S21. The Pourbaix diagram of **Th_3O_2Si**.

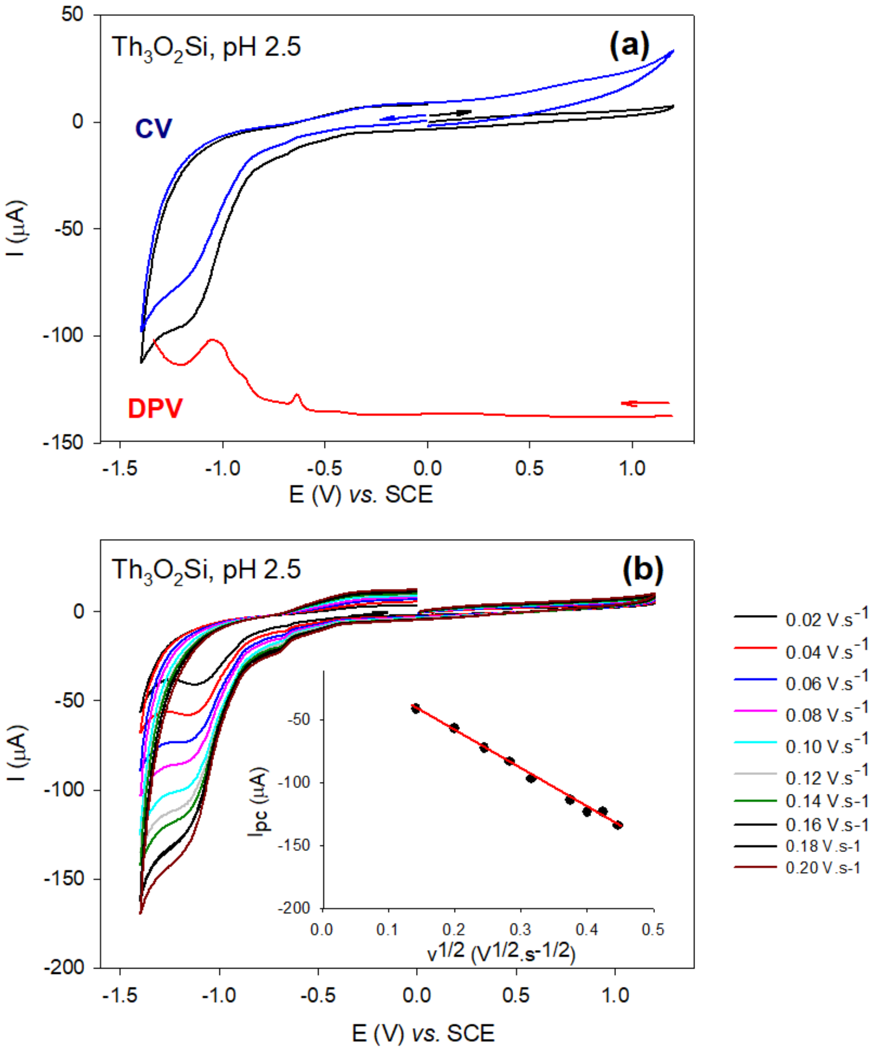

Figure S22. (a) top: cyclic voltammogram (CV) of **Th_3O_3Si** (c = 0.5 mM) in aqueous solution at pH 2.5 containing 0.5 M Na_2SO_4 + H_2SO_4. Working electrode: glassy carbon (GC) disk; auxiliary electrode: Pt wire, reference electrode: SCE. Scan rate: v = 0.1 V/s; bottom: differential pulse voltammetry (DPV) of **Th_3Si** (c = 0.5 mM). (b) cyclic voltammogram of **Th_3Si** measured at different scan rate from 0.02 V/s to 0.20 V/s. Inset: Plot of I_{pc} *vs.* $v^{1/2}$.

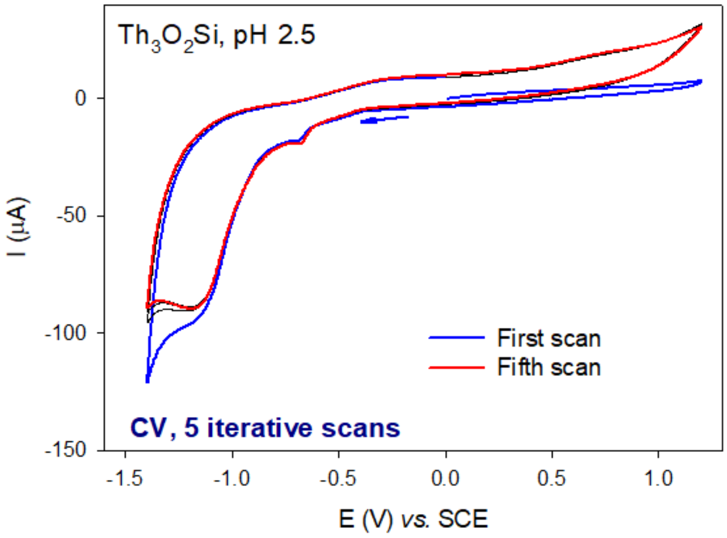

Figure S23. Cyclic voltammogram (CV) of **Th_3O_2Si** (c = 0.5 mM) during 5 iterative cycles in aqueous solution at pH 2.5 containing 0.5 M Na_2SO_4 + H_2SO_4. Working electrode: glassy carbon (GC) disk; auxiliary electrode: Pt wire, reference electrode: SCE. Scan rate: v = 0.1 V/s.

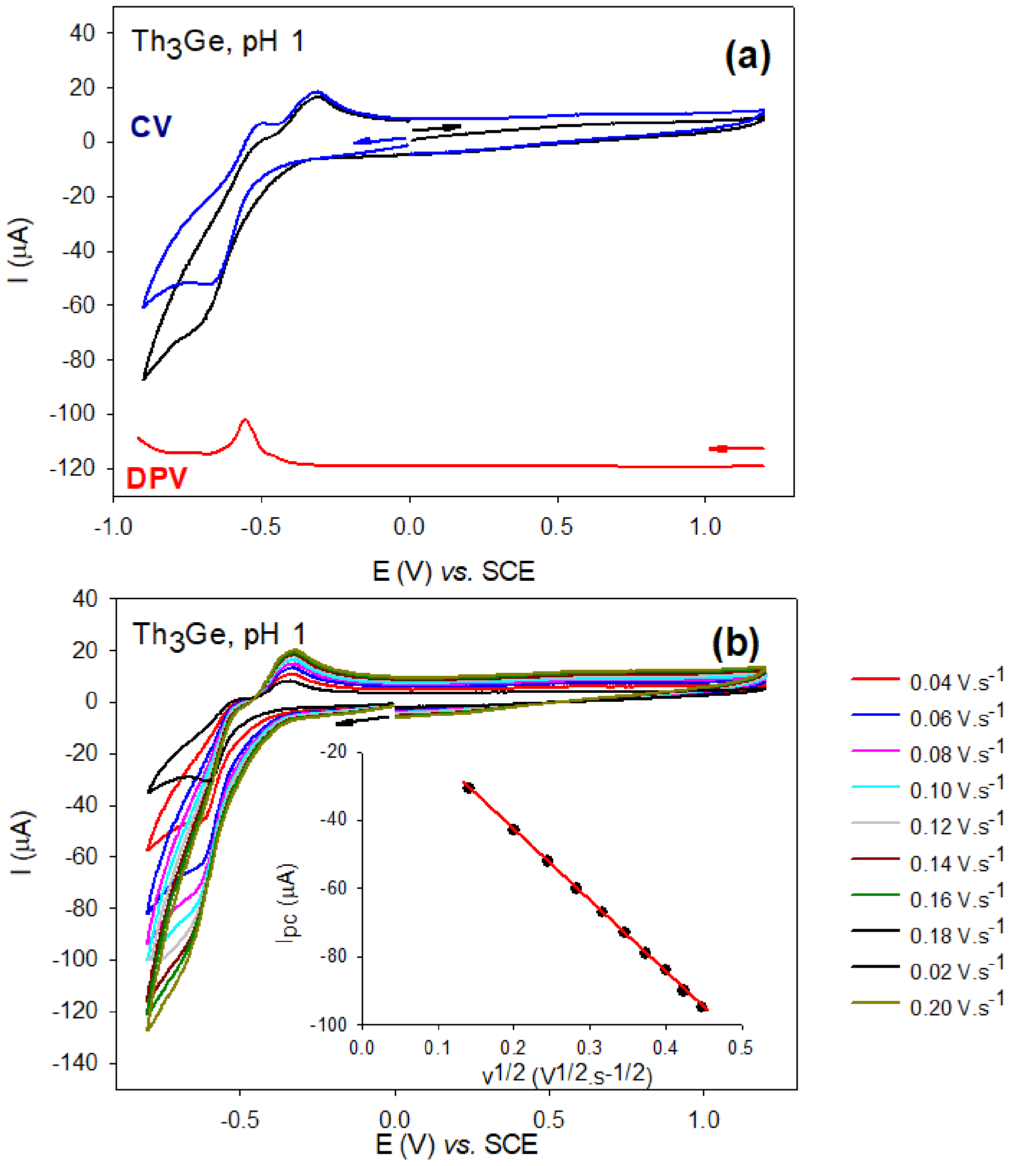

Figure S24. (a) top: cyclic voltammogram (CV) of **Th3Ge** (c = 0.5 mM) in aqueous solution at pH 1 containing 0.5 M Na_2SO_4 + H_2SO_4. Working electrode: glassy carbon (GC) disk; auxiliary electrode: Pt wire, reference electrode: SCE. Scan rate: v = 0.1 V/s; bottom: differential pulse voltammetry (DPV) of **Th3Si** (c = 0.5 mM). (b) cyclic voltammogram of **Th3Si** measured at different scan rate from 0.02 V/s to 0.20 V/s. Inset: Plot of I_{pc} *vs.* $v^{1/2}$.

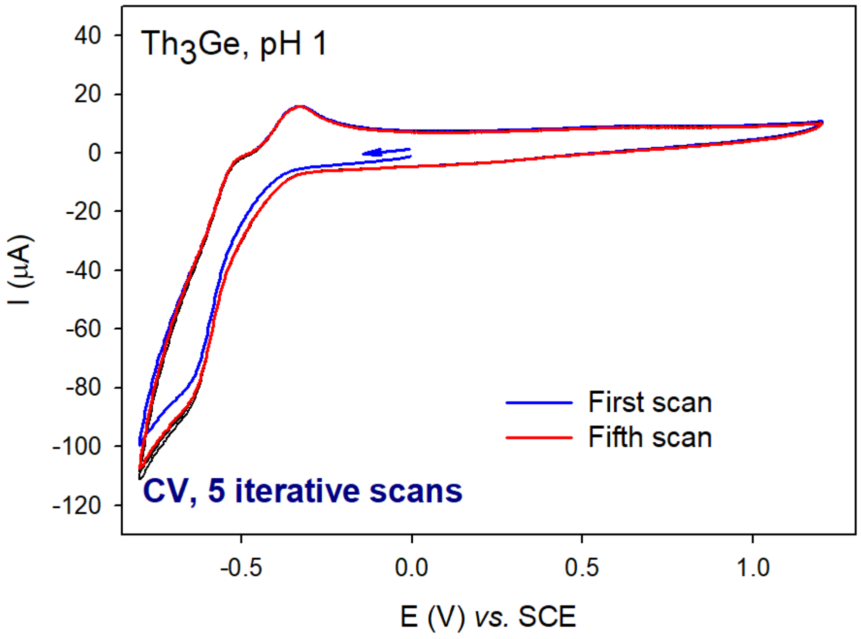

Figure S25. Cyclic voltammogram (CV) of **Th_3Ge** (c = 0.5 mM) during 5 iterative cycles in aqueous solution at pH 1 containing 0.5 M Na_2SO_4 + H_2SO_4. Working electrode: glassy carbon (GC) disk; auxiliary electrode: Pt wire, reference electrode: SCE. Scan rate: v = 0.1 V/s.

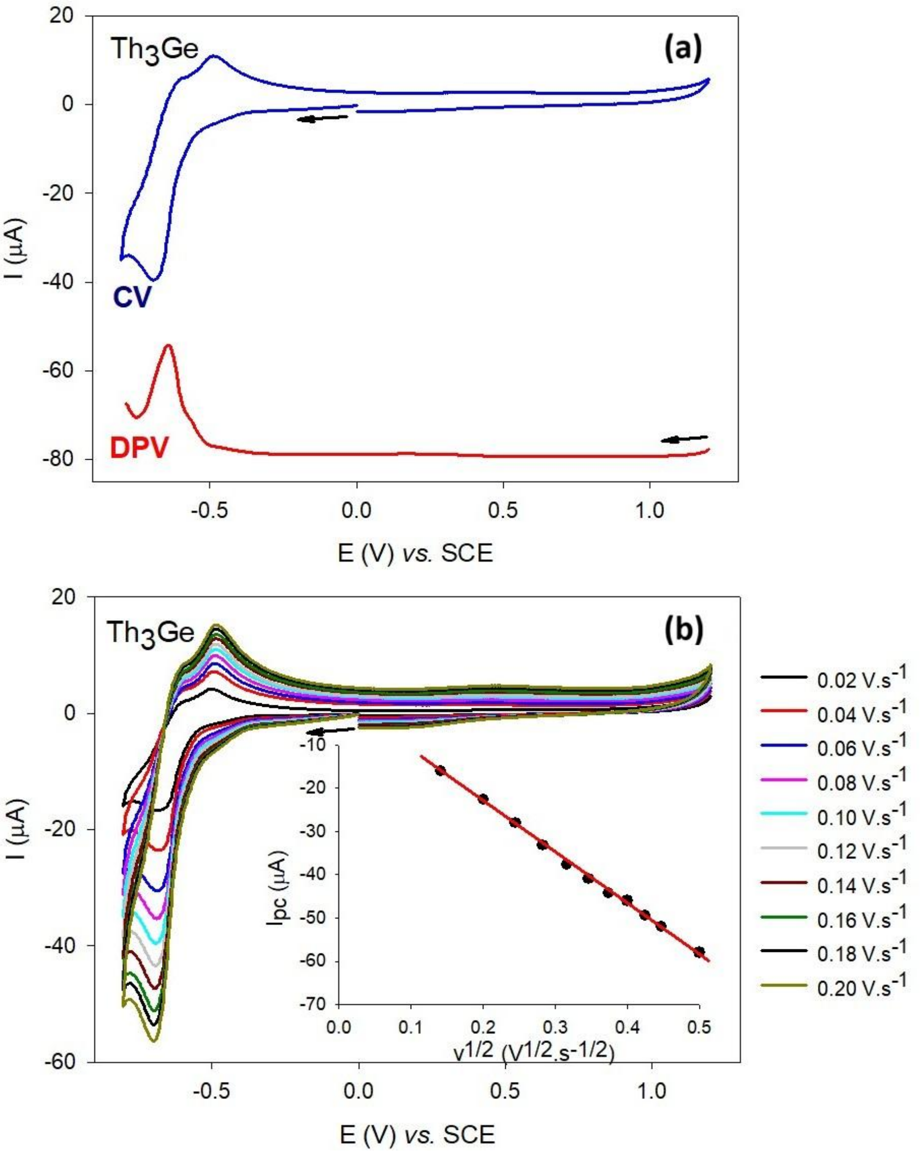

Figure S26. (a) top: cyclic voltammogram (CV) of **Th_3Ge** (c = 0.5 mM) in aqueous solution at pH 2 containing 0.5 M Na_2SO_4 + H_2SO_4. Working electrode: glassy carbon (GC) disk; auxiliary electrode: Pt wire, reference electrode: SCE. Scan rate: v = 0.1 V/s; bottom: differential pulse voltammetry (DPV) of **Th_3Ge** (c = 0.5 mM). (b) cyclic voltammogram of **Th_3Ge** measured at different scan rate from 0.02 V/s to 0.20 V/s. Inset: Plot of I_{pc} *vs.* $v^{1/2}$.

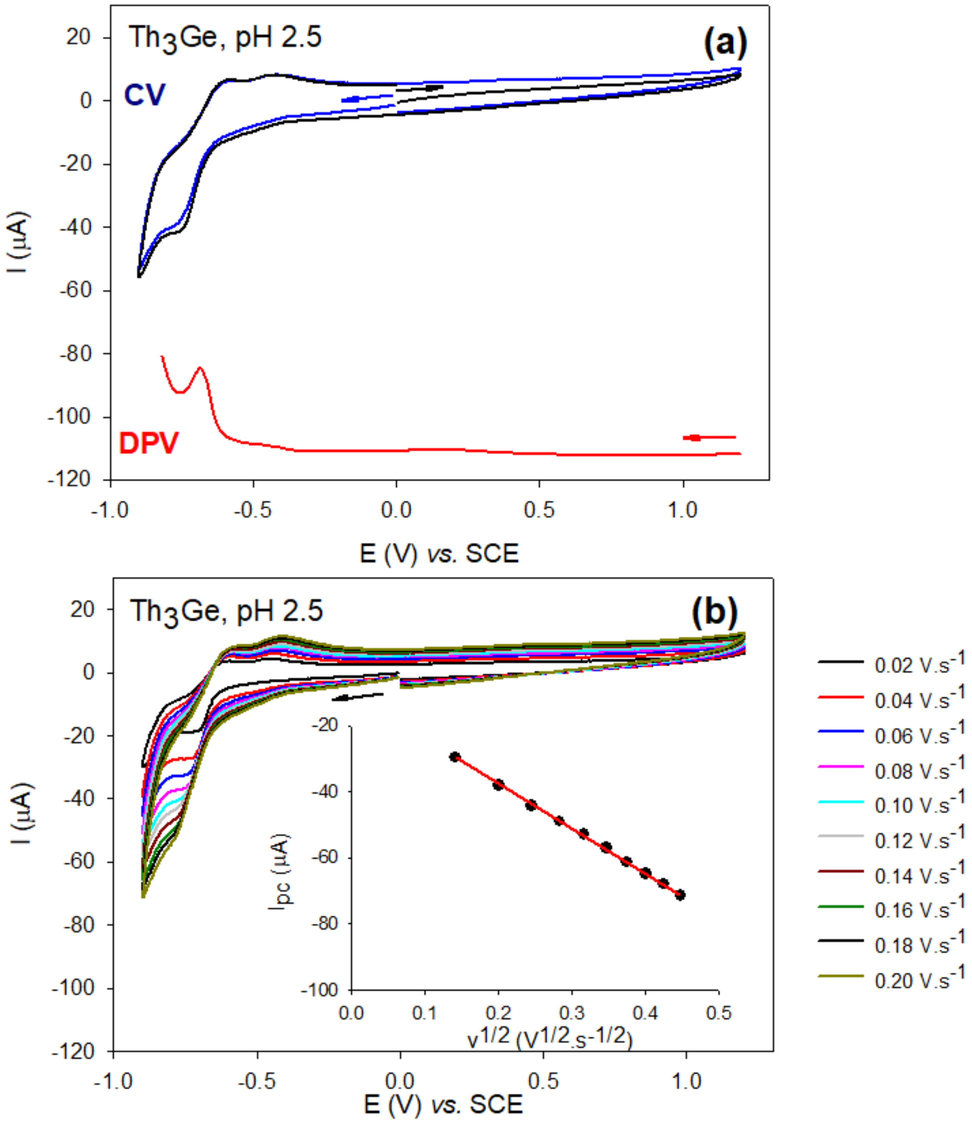

Figure S27. (a) top: cyclic voltammogram (CV) of **Th_3Ge** (c = 0.5 mM) in aqueous solution at pH 2.5 containing 0.5 M Na_2SO_4 + H_2SO_4. Working electrode: glassy carbon (GC) disk; auxiliary electrode: Pt wire, reference electrode: SCE. Scan rate: v = 0.1 V/s; bottom: differential pulse voltammetry (DPV) of **Th_3Si** (c = 0.5 mM). (b) cyclic voltammogram of **Th_3Si** measured at different scan rate from 0.02 V/s to 0.20 V/s. Inset: Plot of I_{pc} *vs.* $v^{1/2}$.

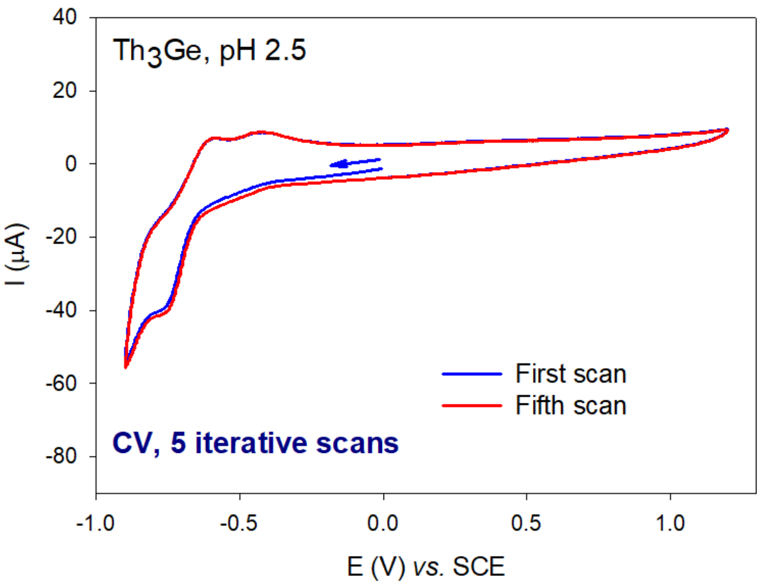

Figure S28. Cyclic voltammogram (CV) of **Th_3Ge** (c = 0.5 mM) during 5 iterative cycle in aqueous solution at pH 2.5 containing 0.5 M Na_2SO_4 + H_2SO_4. Working electrode: glassy carbon (GC) disk; auxiliary electrode: Pt wire, reference electrode: SCE. Scan rate: v = 0.1 V/s.

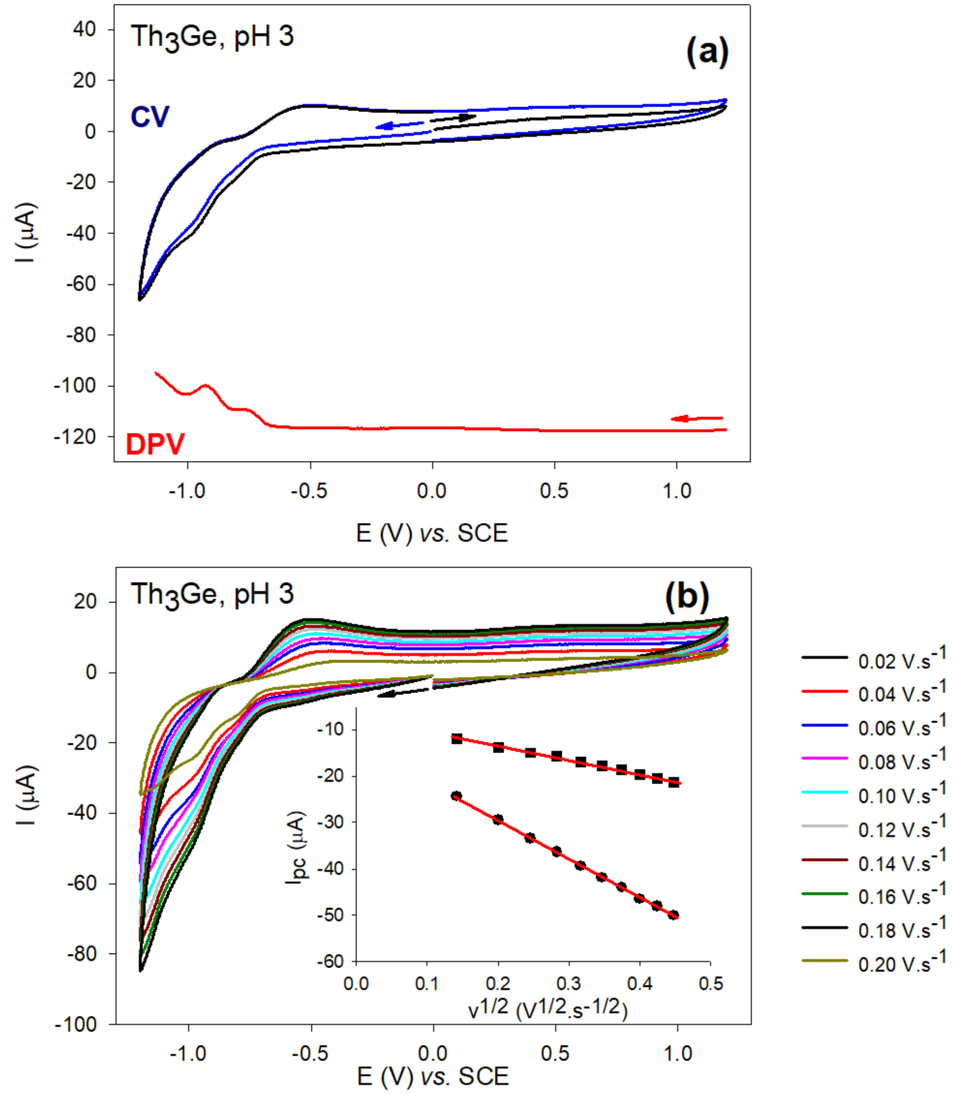

Figure S29. (a) top: cyclic voltammogram (CV) of **Th_3Ge** (c = 0.5 mM) in aqueous solution at pH 3 containing 0.5 M Na_2SO_4 + H_2SO_4. Working electrode: glassy carbon (GC) disk; auxiliary electrode: Pt wire, reference electrode: SCE. Scan rate: v = 0.1 V/s; bottom: differential pulse voltammetry (DPV) of **Th_3Si** (c = 0.5 mM). (b) cyclic voltammogram of **Th_3Si** measured at different scan rate from 0.02 V/s to 0.20 V/s. Inset: Plot of I_{pc} *vs.* $v^{1/2}$.

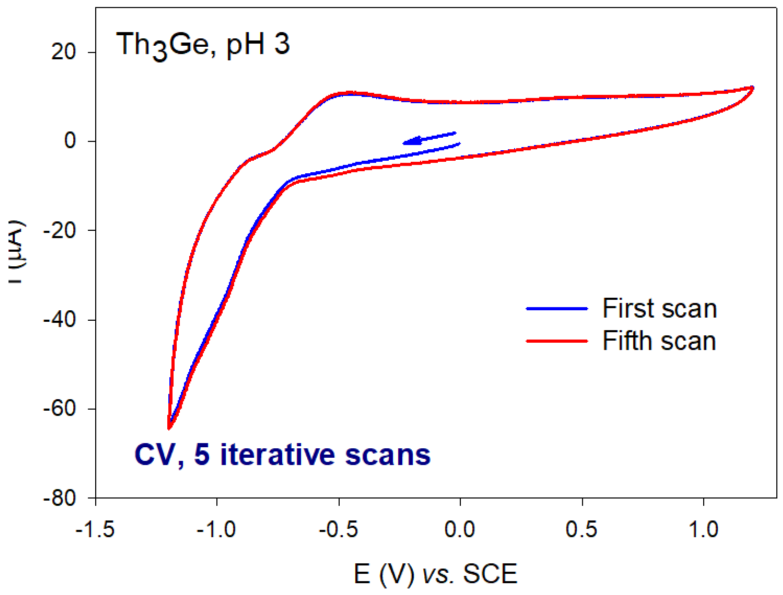

Figure S30. Cyclic voltammogram (CV) of **Th_3Ge** (c = 0.5 mM) during 5 iterative cycles in aqueous solution at pH 3 containing 0.5 M Na_2SO_4 + H_2SO_4. Working electrode: glassy carbon (GC) disk; auxiliary electrode: Pt wire, reference electrode: SCE. Scan rate: v = 0.1 V/s.

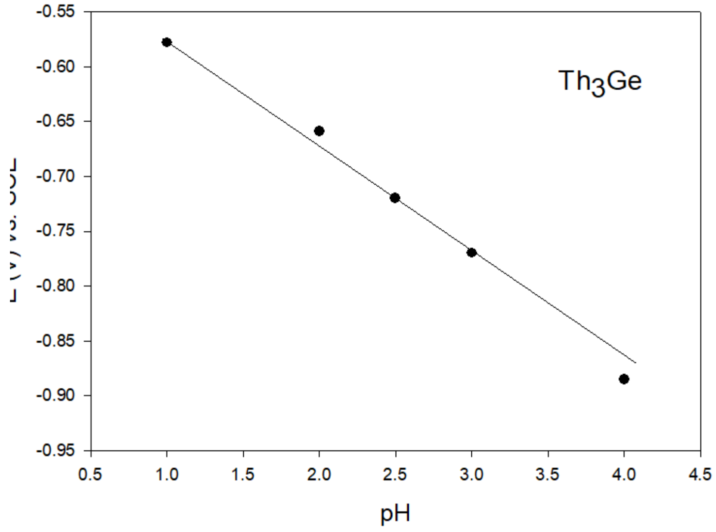

Figure S31. The Pourbaix diagram of **Th_3Ge**.

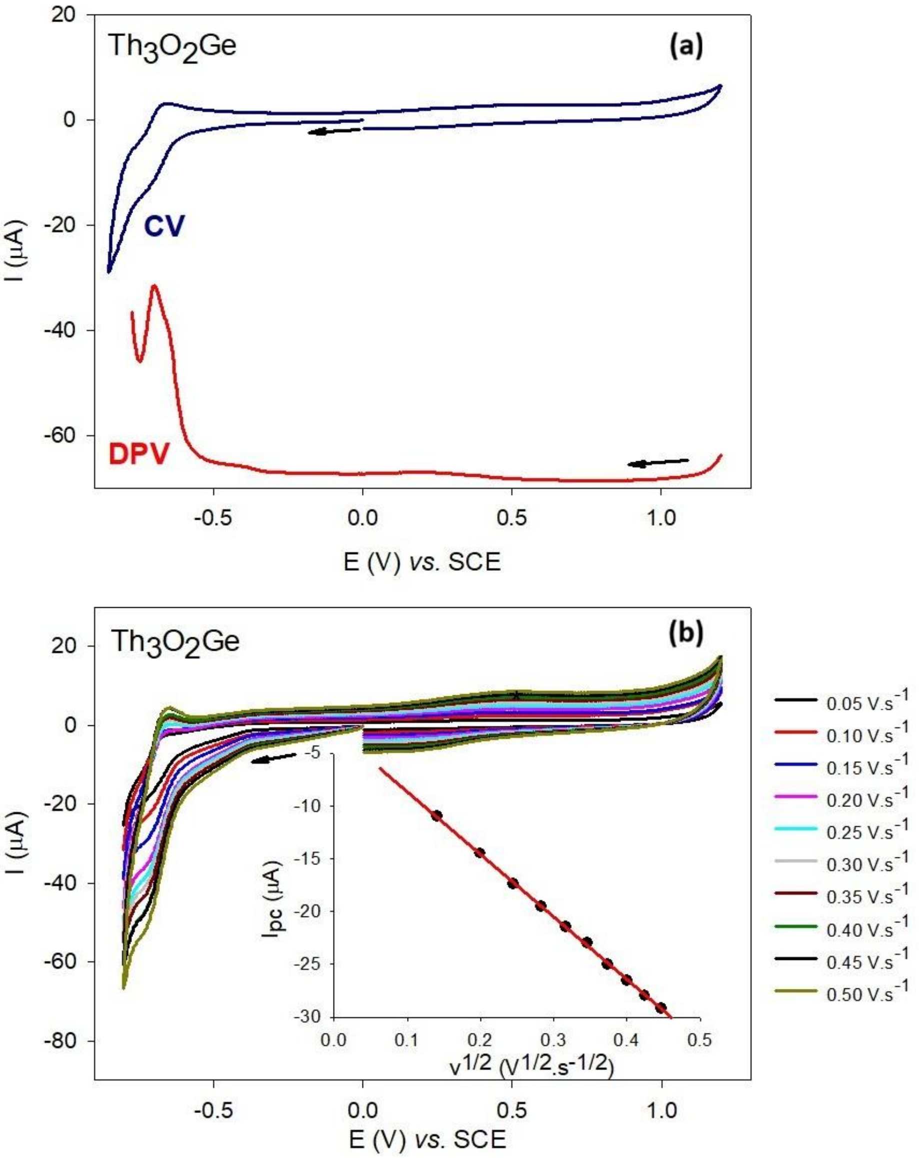

Figure S32. (a) top: cyclic voltammogram (CV) of **Th_3O_2Ge** (c = 0.5 mM) in aqueous solution at pH 2 containing 0.5 M Na_2SO_4 + H_2SO_4. Working electrode: glassy carbon (GC) disk; auxiliary electrode: Pt wire, reference electrode: SCE. Scan rate: v = 0.1 V/s; bottom: differential pulse voltammetry (DPV) of **Th_3O_2Ge** (c = 0.5 mM). (b) cyclic voltammogram of **Th_3O_2Ge** measured at different scan rate from 0.05 V/s to 0.50 V/s. Inset: Plot of I_{pc} *vs.* $v^{1/2}$.

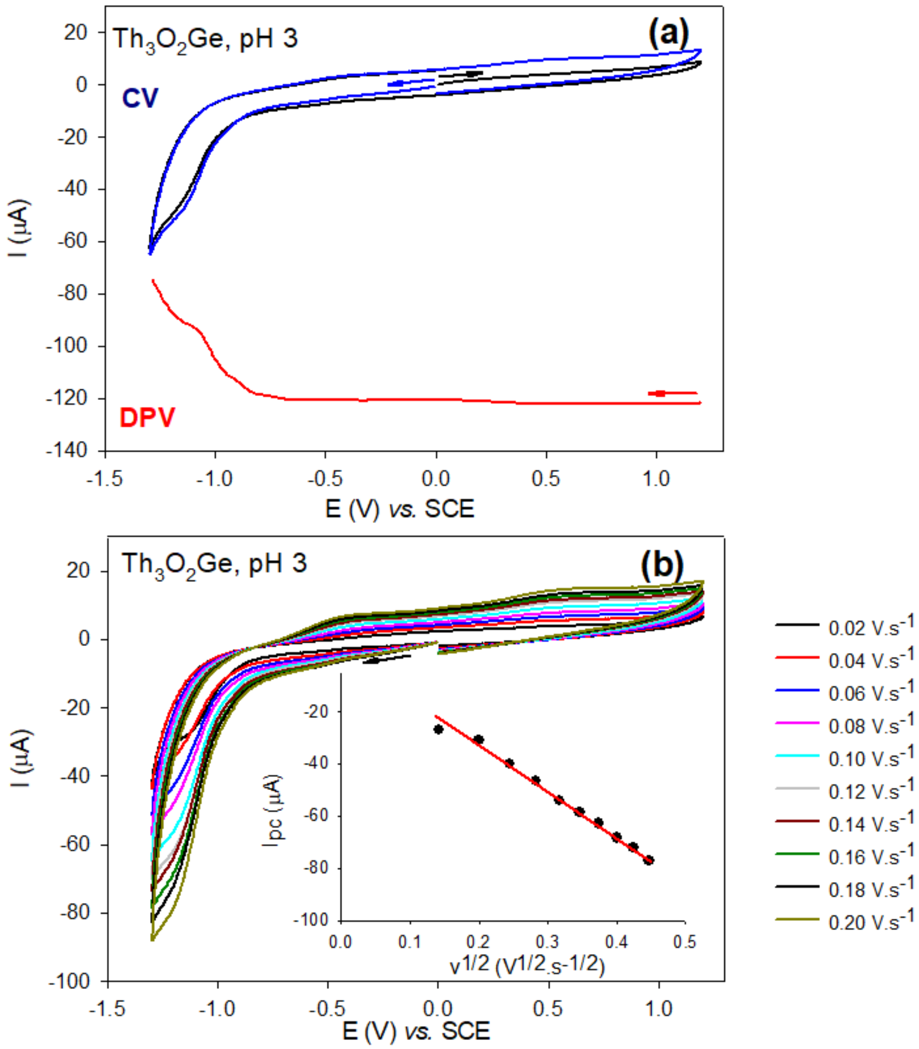

Figure S33. (a) top: cyclic voltammogram (CV) of **Th_3O_3Ge** (c = 0.5 mM) in aqueous solution at pH 3 containing 0.5 M Na_2SO_4 + H_2SO_4. Working electrode: glassy carbon (GC) disk; auxiliary electrode: Pt wire, reference electrode: SCE. Scan rate: v = 0.1 V/s; bottom: differential pulse voltammetry (DPV) of **Th_3Si** (c = 0.5 mM). (b) cyclic voltammogram of **Th_3Si** measured at different scan rate from 0.02 V/s to 0.20 V/s. Inset: Plot of I_{pc} *vs.* $v^{1/2}$.

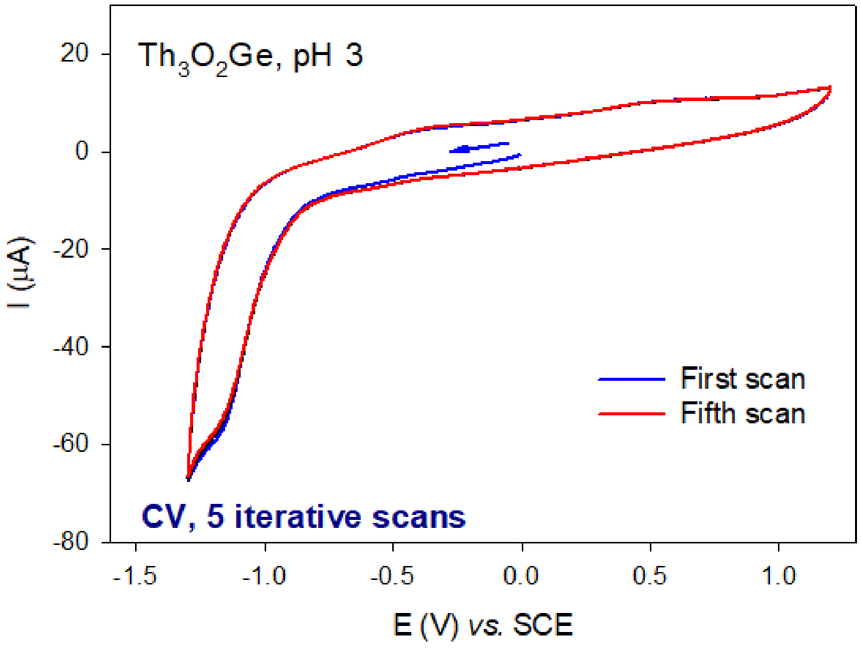

Figure S34. Cyclic voltammogram (CV) of **Th_3O_2Ge** (c = 0.5 mM) during 5 iterative cycles in aqueous solution at pH 3 containing 0.5 M Na_2SO_4 + H_2SO_4. Working electrode: glassy carbon (GC) disk; auxiliary electrode: Pt wire, reference electrode: SCE. Scan rate: v = 0.1 V/s.

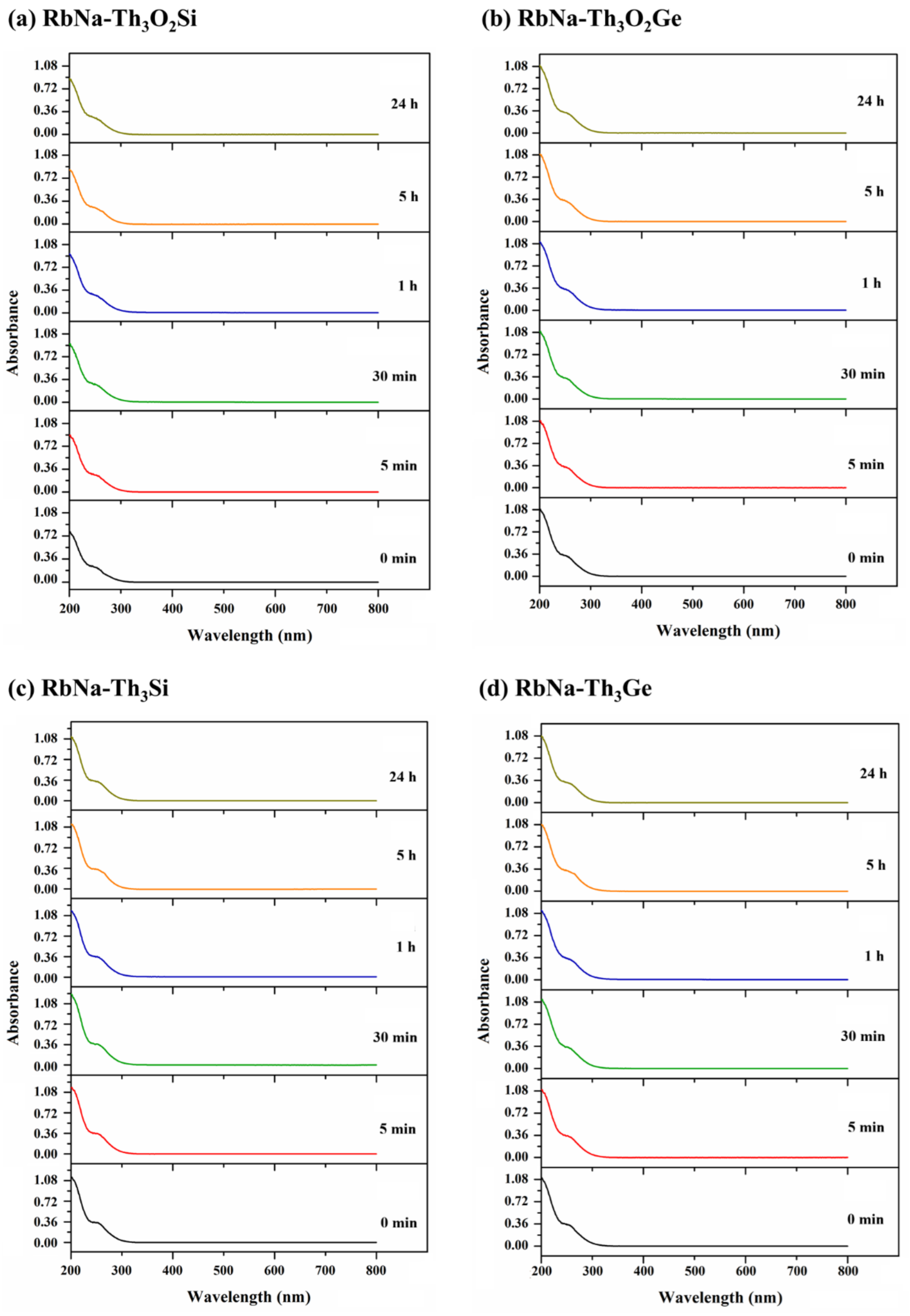

Figure S35. Time-dependent UV-vis absorption spectra of (a) **RbNa-Th_3O_2Si**, (b) **RbNa-Th_3O_2Ge**, (c) **RbNa-Th_3Si**, and (d) **RbNa-Th_3Ge** dissolved in water (6.3 µM) recorded at t = 0 (immediately after preparation), 5 min, 30 min, 1 h, 5 h, and 24 h.

Table S1. Bond valence sum* values for μ_2-bridging and terminal oxygen atoms in the central {$Th_3(\mu_3$-$O_2)(OH)_2(H_2O)_3$} cluster of **RbNa-Th$_3$O$_2$Si**.

Atom	Bond distance (Å)	BVS value	Atom type
O12H	Th1—O12H (2.319)<br>Th2—O12H (2.338)	1.293	Hydroxo
O13H	Th1—O13H (2.348)<br>Th3—O13H (2.347)	1.227	Hydroxo
O23H	Th2—O23H (2.762)<br>Th3—O23H (2.814)	0.374	Aqua
O2TH	Th2—O2TH (2.521)	0.384	Aqua
O3TH	Th3—O3TH (2.539)	0.365	Aqua

* $BVS = \sum_i \exp\left(\frac{R_0 - R_i}{b}\right)$, where R_0 is the empirical bond valence parameter specific to the bond type, R_i is the measured bond length for bond, and b is the empirical constant, typically 0.37 Å.

Table S2. Bond valence sum* values for μ_2-bridging and terminal oxygen atoms in the central {$Th_3(\mu_3$-$O_2)(OH)_2(H_2O)_3$} cluster of **RbNa-Th$_3$O$_2$Ge**.

Atom	Bond distance (Å)	BVS value	Atom type
O12H	Th1—O12H (2.318)<br>Th2—O12H (2.342)	1.288	Hydroxo
O13H	Th1—O13H (2.350)<br>Th3—O13H (2.361)	1.202	Hydroxo
O2W	Th2—O2W (2.779)<br>Th3—O2W (2.808)	0.369	Aqua
O1T2	Th2—O1T2 (2.554)	0.351	Aqua
O1T3	Th3—O1T3 (2.559)	0.347	Aqua

* $BVS = \sum_i \exp\left(\frac{R_0 - R_i}{b}\right)$, where R_0 is the empirical bond valence parameter specific to the bond type, R_i is the measured bond length for bond, and b is the empirical constant, typically 0.37 Å.

Table S3. Bond valence sum* values for μ_2-bridging and terminal oxygen atoms in the central {$Th_3(\mu_3$-$O)(OH)_3(H_2O)$} cluster of **RbNa-Th$_3$Si**.

Atom	Bond distance (Å)	BVS value	Atom type
O12H	Th1—O12H (2.304)<br>Th2—O12H (2.307)	1.375	Hydroxo
O13H	Th1—O13H (2.633)<br>Th3—O13H (2.663)	0.545	Aqua/Hydroxo
O23H	Th2—O23H (2.625)<br>Th3—O23H (2.665)	0.550	Aqua/Hydroxo
O3T3	Th3—O3T3 (2.661)	0.263	Aqua
O123	Th1—O123 (2.309)<br>Th2—O123 (2.335)<br>Th3—O123 (2.287)	2.039	Oxo

* $BVS = \sum_i \exp\left(\frac{R_0 - R_i}{b}\right)$, where R_0 is the empirical bond valence parameter specific to the bond type, R_i is the measured bond length for bond, and b is the empirical constant, typically 0.37 Å.

Table S4. Bond valence sum* values for μ_2-bridging and terminal oxygen atoms in the central {$Th_3(\mu_3$-$O)(OH)_3$}cluster of **RbNa-Th$_3$Ge**.

Atom	Bond distance (Å)	BVS value	Atom type
O1TH	Th1—O1TH (2.399)<br>Th1—O1TH (2.399)	1.068	Hydroxo
O2TH	Th1—O2TH (2.350)<br>Th2—O2TH (2.351)	1.217	Hydroxo
O12H	Th1—O12H (2.270)<br>Th1—O12H (2.270)<br>Th2—O12H (2.314)	2.186	Oxo

* $BVS = \sum_i \exp\left(\frac{R_0 - R_i}{b}\right)$, where R_0 is the empirical bond valence parameter specific to the bond type, R_i is the measured bond length for bond, and b is the empirical constant, typically 0.37 Å.

Table S5. Crystallographic data for mixed potassium-sodium salt of $\mathbf{Th_3O_2Si}$ recrystallized from 1 M lithium acetate solution (pH 4).

Compound	$\mathbf{Th_3O_2Si}$	Z	2
Formula	$[Th_3(O_2)(OH)_2(H_2O)_3(SiW_9O_{34})_2]^{12-}$	D_{calc} (Mg/m^3)	3.481
Crystal system	Triclinic	Absorption coefficient (mm^{-1})	52.04
Space group	$P\bar{1}$	Crystal size (mm)	*0.14 × 0.09 × 0.06*
a (Å)	12.7024(2)	F (000)	4460.0
b (Å)	19.8039(3)	Reflections used [$I > 2\sigma(I)$]	14859
c (Å)	22.8592(5)	Independent reflections	16839
α (°)	64.895(2)	R_{int}	0.174
β (°)	81.891(2)	Goodness-of-fit on F^2	1.08
γ (°)	74.678(2)	R_1 [$I > 2\sigma(I)$][a]	0.08
Volume (Å^3)	5019.32(18)	wR_2[c] (all data)[b]	0.235

Structure of polyanion $\mathbf{Th_3O_2Si}$

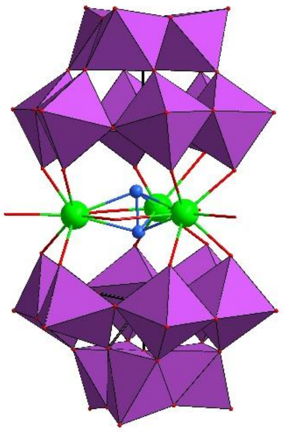

[a] $R_1 = \Sigma||F_o| - |F_c||/\Sigma|F_o|$.

[b] $wR_2 = [\Sigma w (F_o^2 - F_c^2)^2/\Sigma w (F_o^2)^2]^{1/2}$.

Table S6. Crystallographic data for mixed potassium-sodium salt of **Th_3O_2Ge** recrystallized from 1 M lithium acetate solution (pH 4).

Compound	**Th_3O_2Ge**	*Z*	2
Formula	$[Th_3(O_2)(OH)_2(H_2O)_3(GeW_9O_{34})_2]^{12-}$	D_{calc} (Mg/m^3)	3.988
Crystal system	Triclinic	Absorption coefficient (mm^{-1})	28.300
Space group	$P\bar{1}$	Crystal size (mm)	*0.1 × 0.05 × 0.04*
a (Å)	13.3347(5)	F (000)	4662.0
b (Å)	16.6334(6)	Reflections used [$I > 2\sigma(I)$]	9336
c (Å)	23.0289(8)	Independent reflections	15318
α (°)	106.201(3)	R_{int}	0.213
β (°)	93.323(3)	Goodness-of-fit on F^2	1.00
γ (°)	109.260(3)	R_1 [$I > 2\sigma(I)$][a]	0.094
Volume ($Å^3$)	4566.7(3)	wR_2[c] (all data)[b]	0.273

Structure of polyanion **Th_3O_2Ge**

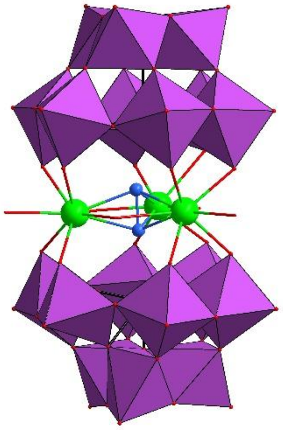

[a] $R_1 = \Sigma||F_o| - |F_c||/\Sigma|F_o|$.
[b] $wR_2 = [\Sigma w (F_o^2 - F_c^2)^2/\Sigma w (F_o^2)^2]^{1/2}$.

Table S7. Crystallographic data for mixed potassium-sodium salt of **Th₃Si** recrystallized from 1 M lithium acetate solution (pH 4).

Compound	**Th_3Si**	Z	1
Formula	$[Th_3(O)(OH)_3(H_2O)(SiW_9O_{34})_2]^{13-}$	D_{calc} (Mg/m^3)	4.361
Crystal system	Triclinic	Absorption coefficient (mm^{-1})	60.589
Space group	$P\bar{1}$	Crystal size (mm)	*0.1 × 0.09 × 0.07*
a (Å)	11.9710	F (000)	5182
b (Å)	12.1084	Reflections used [$I > 2\sigma(I)$]	14423
c (Å)	33.2942	Independent reflections	14838
α (°)	82.803(2)	R_{int}	0.174
β (°)	82.428(2)	Goodness-of-fit on F^2	1.079
γ (°)	73.539(3)	R_1 [$I > 2\sigma(I)$][a]	0.153
Volume (Å^3)	4568.2(2)	wR_2[c] (all data)[b]	0.329

Structure of polyanion **Th_3Si**

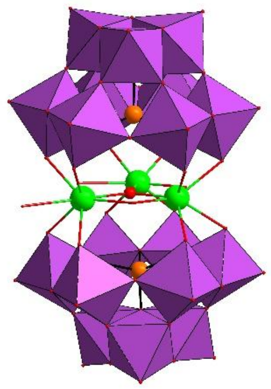

[a] $R_1 = \Sigma||F_o| - |F_c||/\Sigma|F_o|$.

[b] $wR_2 = [\Sigma w (F_o^2 - F_c^2)^2/\Sigma w (F_o^2)^2]^{1/2}$.

Table S8. Crystallographic data for mixed potassium-sodium salt of **Th_3Ge** recrystallized from 1 M lithium acetate solution (pH 4).

Compound	**Th_3Ge**	Z	1
Formula	$[Th_3(O)(OH)_3(GeW_9O_{34})_2]^{13-}$	D_{calc} (Mg/m^3)	3.739
Crystal system	Triclinic	Absorption coefficient (mm^{-1})	55.872
Space group	$P\bar{1}$	Crystal size (mm)	*0.11 × 0.09 × 0.07*
a (Å)	12.1813(2)	F (000)	2242.0
b (Å)	13.5792(2)	Reflections used [$I > 2\sigma$ (I)]	14887
c (Å)	16.0432(3)	Independent reflections	15097
α (°)	72.413(2)	R_{int}	0.114
β (°)	68.631(2)	Goodness-of-fit on F^2	1.76
γ (°)	85.172(2)	R_1 [$I > 2\sigma$ (I)][a]	0.155
Volume ($Å^3$)	2354.79(8)	wR_2[c] (all data)[b]	0.376

Structure of polyanion **Th_3Ge**

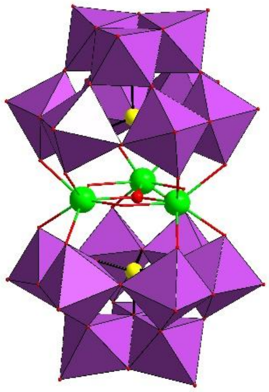

[a] $R_1 = \Sigma||F_o| - |F_c||/\Sigma|F_o|$.
[b] $wR_2 = [\Sigma w (F_o^2 - F_c^2)^2/\Sigma w (F_o^2)^2]^{1/2}$.
